# Distinct and interdependent functions of three RING proteins regulate recombination during mammalian meiosis

**DOI:** 10.1073/pnas.2412961121

**Published:** 2025-01-06

**Authors:** Masaru Ito, Yan Yun, Dhananjaya S. Kulkarni, Sunkyung Lee, Sumit Sandhu, Briana Nuñez, Linya Hu, Kevin Lee, Nelly Lim, Rachel M. Hirota, Rowan Prendergast, Cynthia Huang, Ivy Huang, Neil Hunter

**Affiliations:** ^a^HHMI, University of California, Davis, CA 95616; ^b^Department of Microbiology & Molecular Genetics, University of California, Davis, CA 95616; ^c^Institute for Protein Research, Osaka University, Osaka 565-0871, Japan; ^d^Center for Reproductive Medicine, Clinical Research Center, Shantou Central Hospital, Shantou, China 515041; ^e^Department of Biochemistry & Molecular Biology, Brown University, Providence, RI 02912; ^f^Department of Molecular & Cellular Biology, University of California, Davis, CA 95616

**Keywords:** meiosis, crossover, reproduction, gamete, ubiquitin

## Abstract

During meiosis, each pair of homologous chromosomes becomes connected by at least one crossover, as required for accurate segregation, and adjacent crossovers are widely separated thereby limiting total numbers. In coarsening models, this crossover patterning results from nascent recombination sites competing to accrue a limiting pro-crossover RING-domain protein (COR) that diffuses between synapsed chromosomes. Here, we delineate the localization dynamics of three mammalian CORs in the mouse and determine their interdependencies. RNF212, HEI10, and the newest member RNF212B show divergent spatiotemporal dynamics along synapsed chromosomes, including profound differences in spermatocytes and oocytes, that are not easily reconciled by elementary coarsening models. Contrasting mutant phenotypes and genetic requirements indicate that RNF212B, RNF212, and HEI10 play distinct but interdependent functions in regulating meiotic recombination and coordinating the events of meiotic prophase-I by integrating signals from DNA breaks, homolog synapsis, the cell-cycle, and incipient crossover sites.

## Significance

Meiosis produces haploid gametes by precisely halving the chromosome complement. Crossing over between homologous chromosomes (homologs) is essential for their accurate segregation and defects are associated with infertility, miscarriage, and congenital disease. Factors that ensure crossing over between each pair of homologs include mammalian RING-domain proteins RNF212, HEI10, and RNF212B, alleles of which are linked to infertility and heritable variation in crossover rate. This study focuses on understanding the functions and relationships between these pro-crossover RING proteins (CORs) in the mouse, providing important insights into their roles in regulating recombination, the DNA repair process that produces crossovers. Notably, chromosomal localization dynamics of the three CORs are distinct and show striking sexual dimorphism with important implications for models of crossover control.

During sexual reproduction, parents contribute equally to their offspring by producing gametes with precisely half the normal cellular ploidy. This is achieved via two successive rounds of chromosome segregation during meiosis. Accurate segregation during the first division (MI) requires crossing over between each pair of homologous chromosomes (homologs). In combination with cohesion between sister-chromatids, crossovers create connections called chiasmata that enable stable biorientation of homolog pairs on the meiosis-I spindle (1–3). Defective crossing over can trigger cell death (4), or cause aneuploidy (5, 6), and is therefore associated with infertility, miscarriage, and congenital syndromes (7–10).

Crossing over is typically the minority outcome of meiotic recombination with most initiating DNA double-strand breaks (DSBs) being repaired as noncrossovers without exchange of chromosome arms (11). Also, crossover sites are selected such that each pair of chromosomes obtains at least one, termed crossover assurance; adjacent events are widely and evenly spaced via crossover interference (which also limits total crossovers); and crossover numbers per nucleus show low variance relative to precursor DSBs, reflecting a buffering process known as crossover homeostasis (2, 3, 12). The molecular basis of crossover patterning remains unclear but manifests at the cytological level as selective retention/accumulation of certain pro-crossover factors at designated crossover sites as prophase progresses (2, 3). Among these are RING-domain proteins related to budding yeast Zip3 (13), inferred to mediate modification by SUMO and ubiquitin (2, 14–21). Species differ with respect to the number and subgroup (Zip3/RNF212 and HEI10) of these CrossOver RING proteins (CORs): *Sordariales* and plant genomes encode a single HEI10 homolog (13, 18, 22); *Drosophila* encodes three Zip3/RNF212-like proteins (21, 23); and *C. elegans* has four members with homology to both subgroups (19, 20, 24).

Mammalian genomes encode both RNF212 and HEI10 homologs with interrelated functions that are essential for crossing over (15–17, 25). RNF212 localizes between synapsed chromosomes, initially as numerous foci along the central region of synaptonemal complexes (SCs), before undergoing HEI10-dependent repatterning to accumulate at prospective crossover sites while diminishing elsewhere (15–17). RNF212 and other CORs are inferred to stabilize pro-crossover factors that directly mediate the DNA events of crossing over (15–19, 26, 27). These include the MutSγ complex (MSH4-MSH5) that can bind D-loop and Holliday junction intermediates (28–30), and is inferred to protect them from dissociation by the Bloom-helicase/decatenase complex, BLM-TOPIIIα-RMI1-RMI2 (analogous to Sgs1-Top3-Rmi1 in budding yeast) (31–34). In mouse spermatocytes, MutSγ initially localizes to most recombination sites during late zygotene and early pachytene as homologs complete synapsis (11, 15). Subsequently, RNF212- and HEI10-dependent processes mediate loss of MutSγ from most sites but retention at prospective crossover sites, which then accumulate crossover-specific factors such as the MutLγ endonuclease (2, 29).

A key question for understanding meiotic crossover control is whether crossover sites are specified by the dynamic focal patterning of CORs along SCs, as proposed by recent models that invoke phase-transition/condensation, coarsening, and/or Ostwald ripening processes (19, 35–37). In these models, adjacent recombination sites compete to accumulate a limiting amount of COR protein diffusing along the SC, with crossover designation ensuing at “winning” sites where COR has accrued. Alternatively, COR patterning could occur downstream of crossover patterning via a distinct process, such as mechanical stress (38, 39), in which designation of a crossover site triggers a spreading zone of inhibition that mediates crossover interference. This class of models does not exclude COR coarsening, which could have downstream functions.

Here, we characterize and compare mammalian CORs RNF212, HEI10, and the newest member RNF212B (40–45). Contrasting phenotypes, localization, and genetic dependencies imply that they function as distinct but interdependent modules that integrate signals from DSBs, synapsis, the cell-cycle, and developing crossover complexes to effect spatiotemporal coordination of meiotic prophase. Importantly, the three CORs show diverse spatiotemporal dynamics including striking differences between males and females, features that are not easily reconciled by coarsening models of crossover patterning.

## Results

### *Rnf212b^−/−^* Mice Are Sterile Due to Diminished Crossing Over.

*Rnf212b* null mutants were created via CRISPR-Cas9 (*SI Appendix*, Figs. S1 and S2 and Table S1) and two identical lines, with a frameshift after the eighth codon and premature stop codon after just 27 codons (*SI Appendix*, Fig. S2***B***), were chosen for detailed phenotypic analysis. An independent *Rnf212b* line was recently reported with similar phenotypes to those described below (45).

*Rnf212b^−/−^* males were sterile with testes ~threefold smaller than wild type (WT), cauda epididymides devoid of sperm, and an absence of post metaphase-I spermatocytes (*SI Appendix*, Fig. S3 *A* and *B*). Sterility was also observed for *Rnf212b^−/−^* females, analysis of which is presented in Fig. 6 and *SI Appendix*, Fig. S13.

**Fig. 1.**
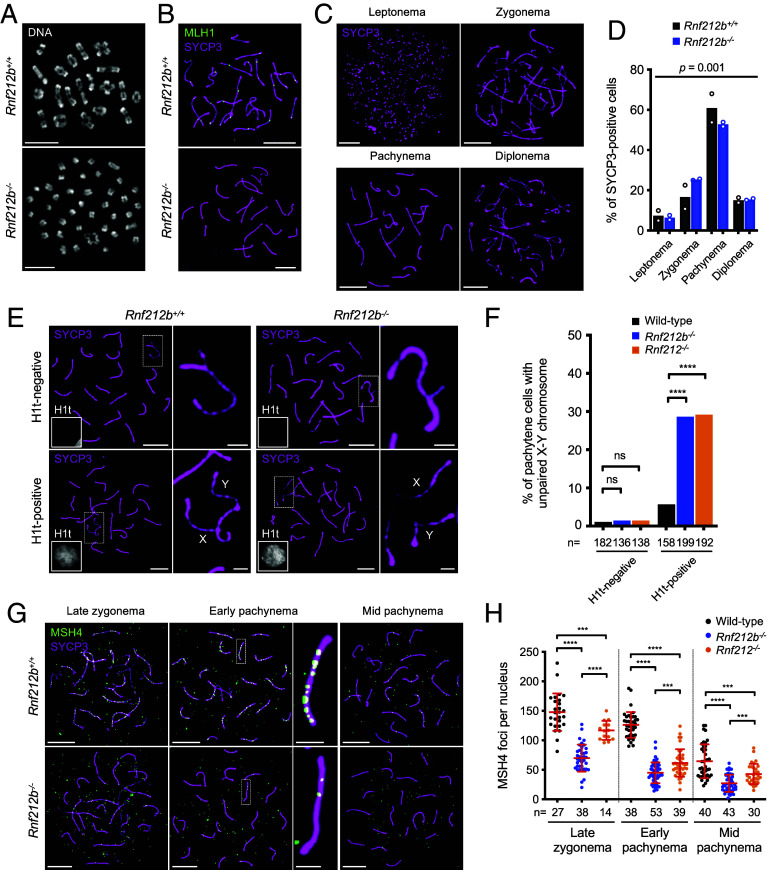
RNF212B is required for crossing over via stabilization of ZMMs in mouse spermatocytes. (*A*) Chiasmata are diminished in *Rnf212b^−/−^* spermatocytes. Diakinesis/metaphase-I stage spermatocytes stained with DAPI. (*B*) MLH1 foci are absent in *Rnf212b^−/−^* spermatocytes. Mid-late pachytene-stage spermatocyte nuclei immunostained for SYCP3 and MLH1. (*C*) Representative images of successive prophase-I stages in chromosome spreads of WT spermatocytes immunostained for SYCP3. (*D*) Distributions of spermatocytes in successive prophase-I stages. Bar graphs indicate means from two mice. *P* = 0.001 for *G* test. >250 unselected SYCP3-positive cells were analyzed per mouse; 578 *Rnf212b^+/+^* and 549 *Rnf212b^−/−^* total cells. (*E*) Autosomal and X-Y synapsis in pachytene-stage spermatocyte nuclei immunostained for SYCP3 and H1t. Magnified images show X-Y chromosomes. (*F*) Premature desynapsis of X-Y chromosomes in *Rnf212b^−/−^* and *Rnf212^−/−^* spermatocytes. ns, not significant (*P* >0.05); ****P ≤0.0001, Fisher’s exact tests. Total numbers of cells analyzed from three mice of each genotype are indicated below the *X* axis. (*G*) Spermatocyte nuclei at indicated stages immunostained for SYCP3 and a ZMM factor MSH4. Representative chromosomes are magnified. (*H*) MSH4 focus counts. Red bars indicate means ± SDs. ***P ≤0.001; ***P ≤0.0001, two-tailed Mann–Whitney tests. n, numbers of nuclei analyzed. (Scale bars, 10 μm for images of full nuclei and 2 μm for magnified panels.)

*Rnf212^−/−^* mutants are sterile because crossover failure results in unconnected univalent chromosomes (15). Analysis of chromosome spreads from diakinesis/metaphase-I spermatocytes showed this is also the case for *Rnf212b^−/−^* mutants (Fig. 1*A*). In WT nuclei, all autosomes presented as bivalents with 24.1 ± 2.3 chiasmata per nucleus (mean ± SD; *n =* 61). Conversely, *Rnf212b^−/−^* nuclei contained primarily univalents with a residual of 1-3 chiasmata (0.66 ± 0.75 chiasmata, *n* = 77). Absence of crossover-specific MLH1 immunostaining foci on pachytene-stage chromosomes (Fig. 1*B*) indicates defects in designation and/or maturation of crossover-specific recombination complexes (confirmed by analyzing two other crossover markers, PRR19 and CDK2; *SI Appendix*, Fig. S3*C*) (16, 46, 47).

### Irregular Chromosome Synapsis in *Rnf212b^−/−^* Mutant Spermatocytes.

To begin to understand why crossing over fails in the *Rnf212b*^−/−^ mutant, progression through prophase-I was assessed by quantifying fractions of spermatocytes at each substage (leptonema, zygonema, pachynema, and diplonema; Fig. 1*C*). Although homologs synapsed in *Rnf212b*^−/−^ spermatocytes, stage distributions indicated overrepresentation of zygotene and fewer pachytene nuclei relative to WT (*P* = 0.001, *G* test), suggesting delayed or unstable synapsis (Fig. 1*D*). Consistent with the latter, while initial pairing and synapsis of the X-Y pseudoautosomal region was efficient, premature desynapsis was elevated >fivefold in mid/late pachytene *Rnf212b^−/−^* spermatocytes (positive for histone variant H1t; Fig. 1 *E* and *F*), analogous to the *Rnf212^−/−^* mutant (15).

### Intermediate Steps of Meiotic Recombination Are Defective in the *Rnf212b^−/−^* Mutant.

The modest synapsis defects of *Rnf212b^−/−^* cells imply that early steps of recombination occur normally, with RNF212B promoting later steps. To test these inferences, pertinent recombination markers were analyzed by immunostaining (Fig. 1 *G* and *H* and *SI Appendix*, Figs. S4 and S5). RAD51 and DMC1 assemble onto DSB ends to form nucleoprotein filaments that catalyze homologous pairing and DNA strand exchange (48). RAD51 and DMC1 focus numbers were slightly elevated in zygotene *Rnf212b^−/−^* spermatocytes (*SI Appendix*, Fig. S4), suggesting mild perturbation of recombination as chromosomes synapse that might reflect the delayed/unstable synapsis inferred above (Fig. 1 *C–F*).

Replication protein A (RPA; comprising RPA1–3) binds single-stranded DNA formed at DSB ends and at D-loops as strand exchange ensues (49). Prominent RPA2 foci emerge in zygonema, as DMC1/RAD51 foci are diminishing, then disappear as DSB repair ensues. Mid-zygotene *Rnf212b^−/−^* and WT spermatocytes formed similar numbers of RPA2 foci, but in subsequent stages, levels were lower in *Rnf212b^−/−^* cells suggesting faster DSB repair (*SI Appendix*, Fig. S5 *A* and *B*). More dramatic reductions were observed for foci of ZMM proteins MSH4, TEX11^Zip4^, and HFM1^Mer3^, with ~threefold fewer foci in the *Rnf212b^−/−^* mutant (Fig. 1 *G* and *H* and *SI Appendix*, Fig. S5 *C*–*F*). The altered dynamics of RPA2 and ZMM foci in *Rnf212b^−/−^* spermatocytes were similar to those seen for *Rnf212^−/−^* (Fig. 1 *G* and *H* and *SI Appendix*, Fig. S5) (15).

### RNF212B Dynamically Localizes to SCs and Recombination Sites.

**Fig. 2.**
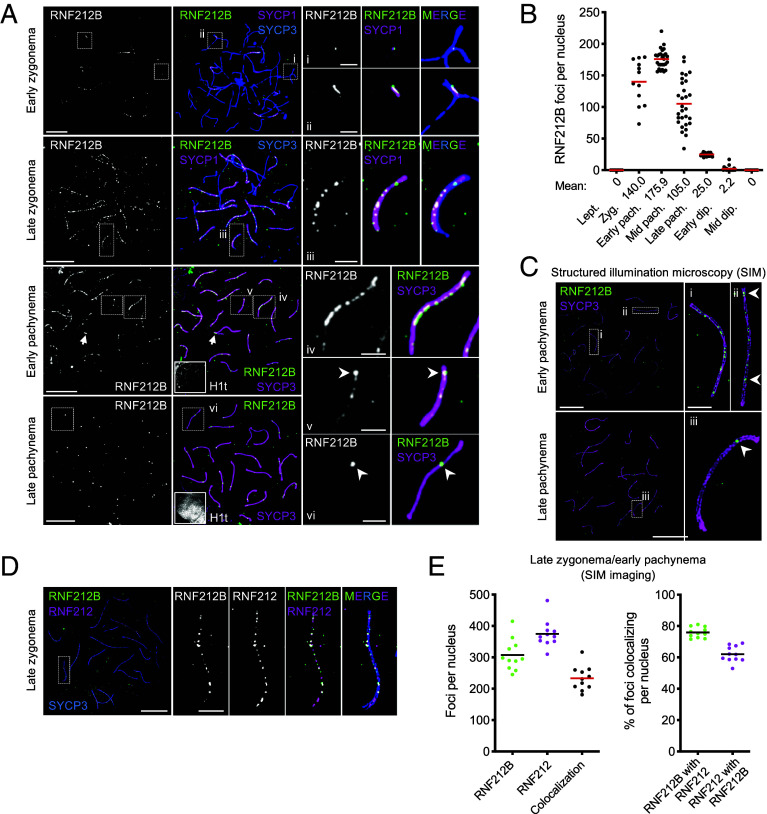
RNF212B localization to SCs and recombination sites in prophase I spermatocytes. (*A*) RNF212B localization at successive prophase I stages in spermatocyte nuclei immunostained for SYCP3, SYCP1, RNF212B, and H1t. Arrowheads highlight amplified RNF212B foci. Arrows indicate synapsed pseudoautosomal region between X-Y chromosomes. (*B*) Numbers of RNF212B foci per nucleus in spermatocytes at successive prophase I stages. Red bars indicate means. Means of focus numbers are shown below the graph. Lept., leptonema; Zyg., zygonema; pach., pachynema; dip., diplonema; numbers of nuclei analyzed were 8, 12, 27, 29, 29, 19, and 11, respectively. (*C*) Localization of RNF212B to the central region of the SC via SIM imaging of early and late pachytene spermatocytes immunostained for SYCP3 and RNF212B. Arrowheads highlight amplified RNF212B foci. (*D*) RNF212B colocalization with RNF212 in SIM image of a late zygotene spermatocyte immunostained for SYCP3, RNF212B, and RNF212. (*E*) Quantification of RNF212B–RNF212 colocalization. *Left*, focus counts. *Right*, degree of colocalization. Black and red bars indicate means. SIM images of 3 late-zygotene and 8 early-pachytene nuclei were analyzed. Magnified images in (*A*), (*C*), and (*D*) show representative chromosomes. (Scale bars, 10 μm for full nuclei and 2 μm for magnified images).

To address whether RNF212B acts locally to stabilize ZMMs at nascent crossover sites, antibodies against RNF212B (isoform a; *SI Appendix*, Fig. S1*B*) were used to immunostain spermatocyte chromosomes (Fig. 2 *A* and *B* and *SI Appendix*, Fig. S6). RNF212B was first detected in very early zygonema, specifically localizing to sites of synapsis initiation marked by SYCP1 (73.1% of short SYCP1 stretches <2 μm colocalized with RNF212B foci; *n* = 38/52 from 11 nuclei). As synapsis ensued, punctate staining extended along SCs, with focus numbers peaking in early pachynema, as synapsis completed, at 175.9 ± 15.2 foci per nucleus (mean ± SD; *n =* 27; Fig. 2*B*). Focus numbers then diminished as a small number of large, bright “amplified” RNF212B foci emerged. By late pachynema, each SC had only one or two large RNF212B foci (25.0 ± 2.3 foci per nucleus; mean ± SD; *n =* 29; Fig. 2*B*). By mid-diplonema, as homologs desynapsed, all RNF212B foci had disappeared. Superresolution structured illumination microscopy (SIM) showed that RNF212B, like RNF212, localizes to the SC central region (Fig. 2*C*).

Phenotypes of *Rnf212b^−/−^* mutants and localization dynamics of RNF212B indicate strong similarities with RNF212. To explore this relationship, colocalization was analyzed by SIM (Fig. 2 *D* and *E*). In late zygotene/early pachytene spermatocytes, RNF212-RNF212B colocalization was high but incomplete with 75.9 ± 3.4% of RNF212B foci colocalizing with RNF212, and 62.1 ± 5.1% of RNF212 foci colocalizing with RNF212B (means ± SDs; *n =* 11 nuclei; Fig. 2 *D* and *E*; note that many more foci are resolved by SIM than by the conventional microscopy used in Fig. 2 *A* and *B* with an average of 307.5 ± 50.0 RNF212B foci, 374.6 ± 43.2 RNF212 foci, and 233.4 ± 38.7 RNF212B–RNF212 cofoci per nucleus).

SIM was also used to analyze localization of RNF212B to recombination sites marked by MSH4 (*SI Appendix*, Fig. S6 *B* and *C*). In early pachynema, RNF212B foci were in twofold excess over MSH4 and only 40.2 ± 6.2% of RNF212B foci colocalized with MSH4 foci; however, 77.2 ± 4.7% MSH4 foci colocalized with RNF212B (means ± SDs; *n =* 7; *SI Appendix*, Fig. S6 *B* and *C*). In late pachynema, numbers of amplified RNF212B foci per nucleus were indistinguishable from those of crossover marker MLH1 and colocalization was essentially absolute (*SI Appendix*, Fig. S6*D*; 99.7 ± 1.0% of RNF212B foci colocalized with MLH1 foci and 99.0 ± 1.7% of MLH1 foci colocalized with RNF212B foci; means ± SDs; *n* = 15 nuclei). Thus, RNF212B foci in late pachytene spermatocytes exclusively localize to crossover sites.

### Amplified RNF212B Foci and HEI10 Designate Crossover Sites in Mid Pachytene Spermatocytes.

**Fig. 3.**
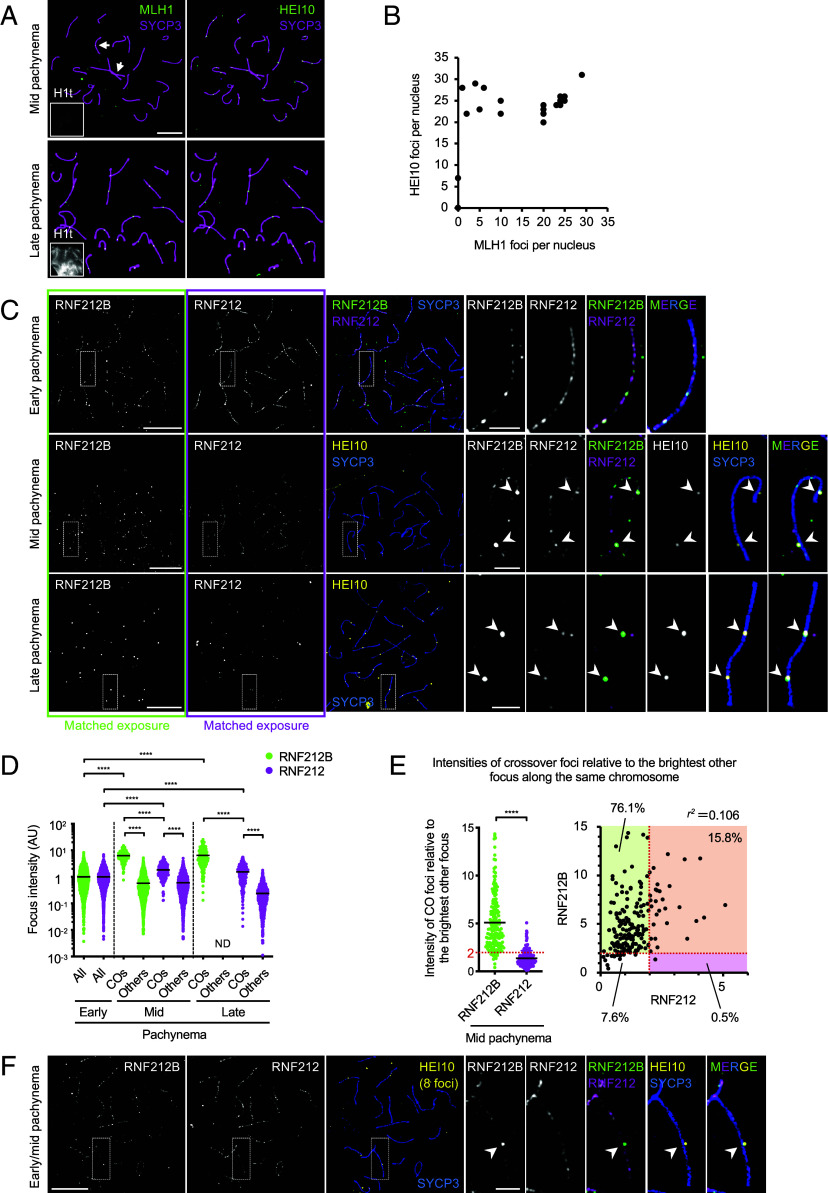
Distinct timing and development of crossover-specific foci of HEI10, RNF212B, and RNF212 (*A*) HEI10 forms crossover foci by mid pachynema. Pachytene spermatocyte nuclei immunostained for SYCP3, HEI10, MLH1, and H1t. *Top*: representative H1t-negative, mid-pachytene nucleus in which small MLH1 foci are emerging on just a few chromosomes (indicated by arrows) whereas bright HEI10 foci are present on all autosomes. *Bottom*: representative H1t-positive late-pachytene nucleus in which both MLH1 and HEI10 form clear, overlapping crossover-specific foci on all autosomes. (*B*) Quantification of HEI10 and MLH1 foci in pachytene spermatocytes confirms that MLH1 foci develop only in nuclei with a full complement of HEI10 foci. 25 pachytene nuclei were randomly selected and analyzed. (*C*) Amplification of RNF212B at prospective crossover sites is stronger than RNF212. Airyscan images of early, mid and late pachytene spermatocyte nuclei immunostained for SYCP3, RNF212B, RNF212, and HEI10. In all cases, RNF212B and RNF212 image exposures are matched. Arrowheads indicate crossover sites marked by HEI10. (*D*) Quantification of focus intensities for RNF212B and RNF212. Black bars indicate means. ****P ≤0.0001, two-tailed Mann–Whitney tests. 8 nuclei at each stage were analyzed by Airyscan imaging. Total numbers of foci analyzed: 2,449 RNF212B foci and 2,513 RNF212 foci in early pachynema; 194 crossover foci, 2,004 other RNF212B foci and 2,291 other RNF212 foci in mid pachynema; 206 crossover foci and 988 other RNF212 foci in late pachynema. All, all foci; COs, crossover foci colocalized with HEI10 foci; Others, foci that do not colocalize with HEI10 foci. ND, not detected. Quantification of focus intensities in representative individual nuclei in (*D*) is shown in *SI Appendix*, Fig. S7*A*. (*E*) Per chromosome analysis for autosomes in mid-pachytene spermatocyte nuclei. Intensity of each crossover-associated RNF212B or RNF212 focus relative to that of the brightest other (non-crossover) focus along the same chromosome plotted separately (*Left*) and in two dimensions (*Right*). Red dashed lines indicate a relative focus intensity of 2. Detailed representation of a per chromosome analysis is shown in *SI Appendix*, Fig. S7*B*. ****P ≤0.0001, two-tailed Mann–Whitney tests. 186 crossover foci from 8 mid-pachytene nuclei were analyzed. Two crossover-associated RNF212B foci with no other RNF212B foci along the same chromosome were excluded from the plots. (*F*) RNF212B differentiation without detectable RNF212 differentiation during early to mid pachynema. Airyscan images of an early to mid-pachytene spermatocyte nucleus, with 8 HEI10 foci, immunostained for SYCP3, RNF212B, RNF212, and HEI10. Arrowheads indicate crossover sites marked by HEI10. Magnifiedimages in (*C*) and (*F*) show representative chromosomes. (Scale bars, 10 μm for full nuclei and 2 μm for magnified images.)

Unlike RNF212B and RNF212, numerous foci of HEI10 were not detected in zygotene or early pachytene spermatocytes. Instead, HEI10 foci emerged in mid pachynema already with a crossover-specific pattern, i.e. crossover designation has occurred by this stage (Fig. 3 *A* and *B*) (16, 17). Colocalizing foci of MLH1 appeared later, in nuclei with a full complement of HEI10 foci.

Having defined HEI10 foci as the earliest detectable marker of crossover designation to date, at least in spermatocytes (for oocytes; see Fig. 7) we set out to determine their spatial-temporal relationship with amplification of RNF212B and RNF212 foci. Focus intensities were measured from images with matched exposures and normalized to mean intensities in early pachytene nuclei lacking HEI10 foci (Fig. 3 *C* and *D* and *SI Appendix*, Fig. S7*A*). In mid-pachytene nuclei with ≥ 1 HEI10 crossover focus per autosomal SC, colocalizing RNF212B foci were 11-fold brighter than other RNF212B foci in the same nuclei (6.29 ± 2.66 for 194 crossover foci versus 0.57 ± 0.59 for 2,004 other foci from 8 nuclei; means ± SDs; *P* < 0.0001, Mann–Whitney test) and 6.3-fold brighter than foci in early pachytene, consistent with the possibility that crossover foci grow at the expense of other foci. By comparison, crossover-associated RNF212 foci were only threefold brighter than other foci in the same nuclei (1.83 ± 1.05 for 194 crossover foci versus 0.60 ± 0.58 for 2,291 other foci form 8 nuclei; means ± SDs; *P* < 0.0001, Mann–Whitney test) and 1.8-fold brighter than those in early pachytene. Thus, at designated crossover sites in mid pachytene, RNF212B shows stronger amplification than RNF212.

In late pachytene spermatocytes, remaining RNF212B foci were crossover specific and had intensities comparable to crossover foci in mid-pachytene (6.42 ± 4.84-fold brighter than early pachytene foci; 206 crossover foci from 8 nuclei; mean ± SD) implying that RNF212B foci do not continue to enlarge between mid and late pachytene, i.e. RNF212B is lost from other sites as opposed to being absorbed into crossover foci. By contrast, RNF212 staining in late pachytene nuclei comprised a mixture of crossover foci and other small residual foci. Crossover-associated RNF212 foci were sixfold brighter than other foci in the same nuclei (1.53 ± 1.13 for 206 crossover foci versus 0.24 ± 0.23 for 988 other foci from 8 nuclei; means ± SDs; *P* < 0.0001, Mann–Whitney test) but only 1.5-fold brighter than those in early pachytene. Thus, crossover-associated RNF212 foci continue to differentiate between mid and late pachynema but this may occur primarily via loss of RNF212 from noncrossover sites as opposed to continued growth of crossover foci. Consistent with these inferences, total signal intensity of RNF212B per nucleus was unchanged from early to mid pachynema, but then decreased from mid to late pachynema as RNF212B was lost from noncrossover sites; by comparison, total signal intensity of RNF212 reduced during both early to mid, and mid to late pachynema (1.00 ± 0.21, 0.97 ± 0.24, and 0.54 ± 0.33 for RNF212B; and 1.00 ± 0.32, 0.69 ± 0.31, and 0.22 ± 0.10 for RNF212 in early, mid, and late pachynema, respectively, *n* = 8 nuclei each; *SI Appendix*, Fig. S7*C*).

Per chromosome analysis in mid pachytene highlighted additional distinctions (Fig. 3*E* and *SI Appendix*, Fig. S7*B*). RNF212B foci at crossover sites were on average 5.1-fold brighter relative to the brightest other focus along the same chromosome, whereas RNF212 foci were only 1.4-fold brighter (Fig. 3 *E*, *Left*). When relative intensities were plotted for individual pairs of colocalizing RNF212-RNF212B crossover foci, almost no correlation was detected (*r^2^* = 0.106), i.e. at mid pachynema, bright RNF212B foci are not coincident with bright RNF212 foci (Fig. 3 *E*, *Right*).

In mid-pachynema, 76.1% of designated crossover sites (HEI10 foci; *n =* 140/184, 8 nuclei) showed amplification for RNF212B (≥ twofold brighter than the brightest other focus along the same chromosome) but not for RNF212. Oppositely only 0.5% of crossover sites (*n =* 1/184; 8 nuclei) showed amplification of only RNF212 but not RNF212B (Fig. 3 *E*, *Right*). Thus, crossover-specific amplification of RNF212B occurs earlier than RNF212. This was most pronounced as HEI10 foci were emerging; in nuclei with <19 HEI10 foci, RNF212B showed clear amplification at designated crossover sites but RNF212 did not (Fig. 3*F*).

Together, focus dynamics in spermatocytes reveal that RNF212B foci amplify at designated crossover sites during early-to-mid pachytene, coincident with the appearance of HEI10 foci (*SI Appendix*, Fig. S8); then RNF212B is lost from other sites without additional amplification of crossover foci. By contrast, RNF212 crossover foci differentiate later, during mid-to-late pachytene; and loss of RNF212 from other sites, rather than growth, may be a major cause of changes in relative intensity.

### Interdependence Between HEI10, RNF212B, and RNF212.

**Fig. 4.**
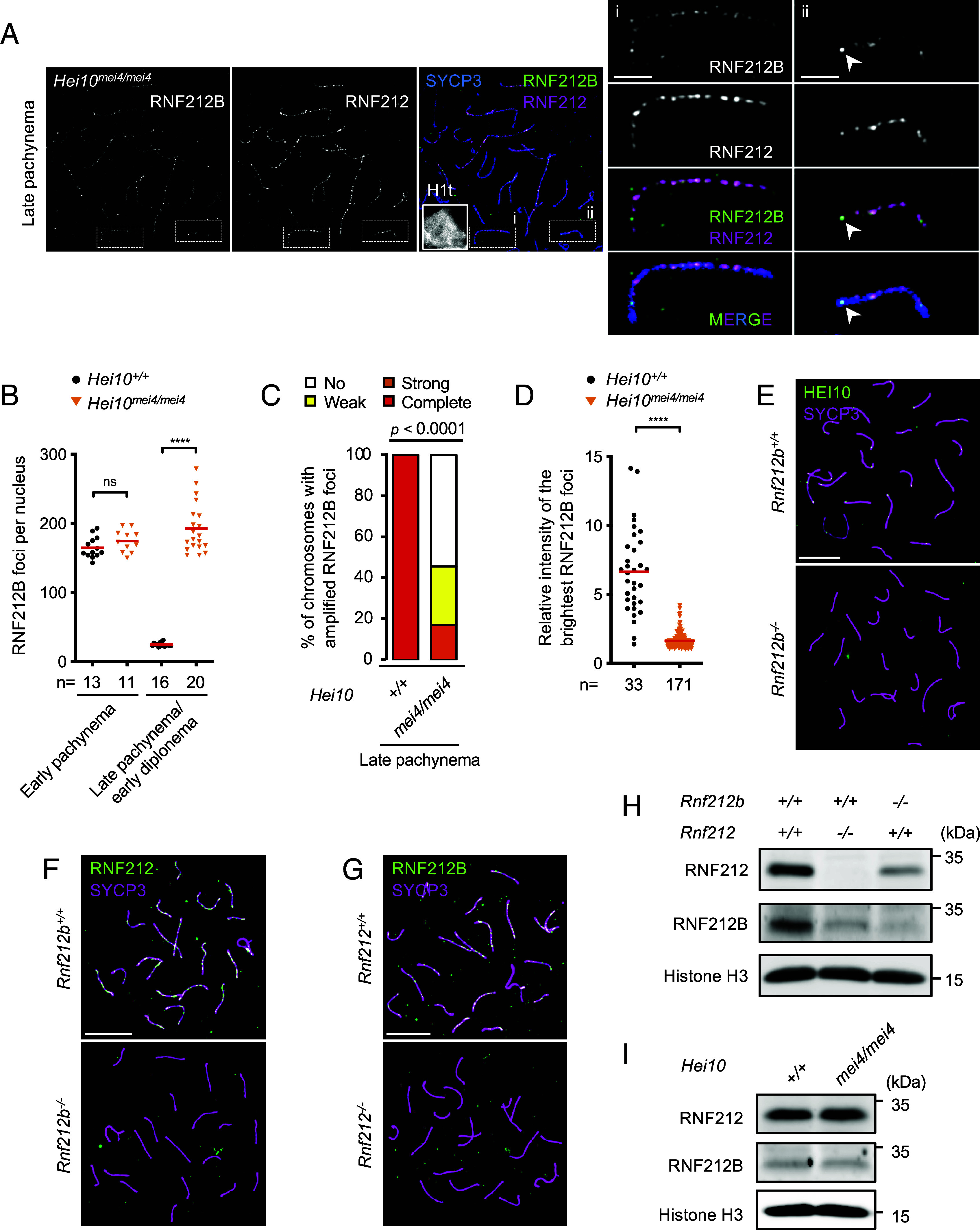
Interdependence between HEI10, RNF212, and RNF212B (*A*) Persistent RNF212B staining in *Hei10^mei4/mei4^* spermatocytes. Airyscan images of a late-pachytene nucleus immunostained for SYCP3, RNF212B, RNF212, and H1t. Magnified panels show representative chromosomes with: i) no apparent differentiation of RNF212B; or ii) some differentiation, indicated by arrowheads. (Scale bars, 10 μm in images of full nuclei and 2 μm in the magnified panels). (*B*) Focus counts of RNF212B. Red bars indicate means. Total numbers of nuclei analyzed are indicated below the *X*-axis. For late stages, only late pachytene nuclei were analyzed for *Hei10^+/+^* whereas both late pachytene and early diplotene nuclei were analyzed for *Hei10^mei4/mei4^* because of the early desynapsis of *Hei10^mei4/mei4^* cells (16). ns, not significant (*P* >0.05); ****P ≤0.0001, two-tailed Mann–Whitney tests. (*C*) Differentiation of RNF212B foci. Autosomes in late-pachytene *Hei10^+/+^* and *Hei10^mei4/mei4^* spermatocyte nuclei were assigned to four classes based on the degree of RNF212B differentiation. Strong, weak, or no differentiation represents autosomes where the brightest RNF212B foci were respectively ≥twofold, 1.5 to 2-fold, or <1.5-fold brighter relative to the third brightest focus along the same SC. Complete differentiation defines autosomes that have only one or two bright RNF212B foci. 8 late-pachytene *Hei10^+/+^* and 9 late-pachytene *Hei10^mei4/mei4^* nuclei were analyzed by Airyscan imaging. *P* <0.0001, *G* test. (*D*) Intensity of the brightest RNF212B foci relative to the third brightest focus along the same chromosome in late pachytene *Hei10^mei4/mei4^* and mid pachytene *Hei10^+/+^* spermatocyte nuclei. Red bars indicate means. Nuclei analyzed were the same as in (*C*) and Fig. 3*E*, respectively, but chromosomes with two crossover foci were analyzed for *Hei10^+/+^* spermatocytes. Total numbers of foci analyzed are indicated below the *X*-axis. ****P ≤0.0001, two-tailed Mann–Whitney test. (*E*) Crossover-specific HEI10 foci require RNF212B. Mid/late pachytene nuclei from WT and *Rnf212b^−/−^* spermatocytes immunostained for SYCP3 and HEI10. (Scale bars, 10 μm.) (*F* and *G*) RNF212 (*F*) and RNF212B (*G*) are interdependent for chromosome localization. Early pachytene spermatocyte nuclei were immunostained for SYCP3 and RNF212 (F) or RNF212B (*G*). (Scale bars, 10 μm.) (*H*) Interdependent stability of RNF212B and RNF212. Testis extracts from juveniles (16 to 18 dpp) immunoblotted for RNF212 and RNF212B. Histone H3 is a loading control. (*I*) RNF212B and RNF212 are stable in the absence of HEI10. Immunoblotting for RNF212, RNF212B, and Histone H3 as in (*H*).

Our previous studies evoked a model in which RNF212 establishes a precondition for crossover/noncrossover differentiation by stabilizing nascent intermediates and stalling recombination by rendering progression dependent on HEI10 (16, 17). Notably, in *Hei10^mei4/mei4^* mutant spermatocytes, abundant RNF212 foci persist along synapsed chromosomes and recombination stalls at an intermediate step marked by persistence of ZMM foci (16, 17). Similarly, initial RNF212B staining in early pachytene spermatocytes was indistinguishable from WT but abundant foci persisted along SCs until early diplonema (Fig. 4 *A* and *B*). Moreover, amplified RNF212B foci were not detected along most chromosomes (Fig. 4 *C* and *D*). Specifically, for 54.4% of chromosomes from late-pachytene *Hei10*^*mei4/mei4*^ spermatocytes (with strong H1t staining), neither the first nor second brightest focus was ≥1.5-fold brighter than the third-brightest focus along the same chromosome, and only 17.0% of chromosomes had RNF212B foci ≥ twofold brighter than the third brightest focus (*n* = 93/171 and 29/171, respectively; 9 nuclei; Fig. 4 *A* and *C*). The most amplified RNF212B foci were on average only 1.6-fold brighter (with a maximum of 4.2-fold) relative to the third brightest focus; whereas RNF212B crossover foci in WT mid-pachytene nuclei were on average 6.7-fold brighter relative to the brightest other focus (Fig. 4*D*; 33 WT chromosomes with two crossover foci were used for comparison to *Hei10*^*mei4/mei4*^; 8 nuclei). Thus, crossover-specific patterning of RNF212B is dependent on HEI10. Reciprocally, immunostaining for HEI10 in *Rnf212b^−/−^* spermatocytes revealed that HEI10 foci are dependent on RNF212B (Fig. 4*E*).

Our localization analysis is incompatible with RNF212B and RNF212 functioning as an obligate heterocomplex of fixed stoichiometry. Even so, immunostaining in *Rnf212b^−/−^* and *Rnf212^−/−^* mutants indicated an interdependent relationship. RNF212 and RNF212B foci were diminished in *Rnf212b^−/−^* and *Rnf212^−/−^* meiocytes, respectively (Fig. 4 *F* and *G* and *SI Appendix*, Fig. S9 *A* and *B*). Immunoblotting revealed levels of RNF212 and to a greater extent RNF212B were reduced in testis extracts from *Rnf212b^−/−^* and *Rnf212^−/−^* mutants, respectively (Fig. 4*H*); by contrast levels were unaffected by *Hei10^mei4/mei4^* mutation (Fig. 4*I*).

RNF212B–RNF212 interaction was suggested by yeast two-hybrid assay (Y2H), and self-interaction of both proteins was also inferred, although RNF212 self-interaction appeared to be much weaker than that of RNF212B (*SI Appendix*, Fig. S9 *C* and *D*). Thus, RNF212B and RNF212 may physically interact and are mutually dependent for protein stability and thus chromosomal localization. Additional analysis showed that an intact RING finger domain was essential for RNF212B self-interaction by Y2H, for the stability of RNF212B and RNF212 in vivo, and thus for the pro-crossover function of RNF212B (*SI Appendix*, Figs. S10 and S11). Y2H interaction between HEI10 and RNF212B (but not HEI10 and RNF212) was also detected (*SI Appendix*, Fig. S9 *C* and *D*), which might reflect their spatiotemporal colocalization at designated crossover sites.

### Distinct Regulation of RNF212B and RNF212 by Recombination and Synaptonemal Complex.

**Fig. 5.**
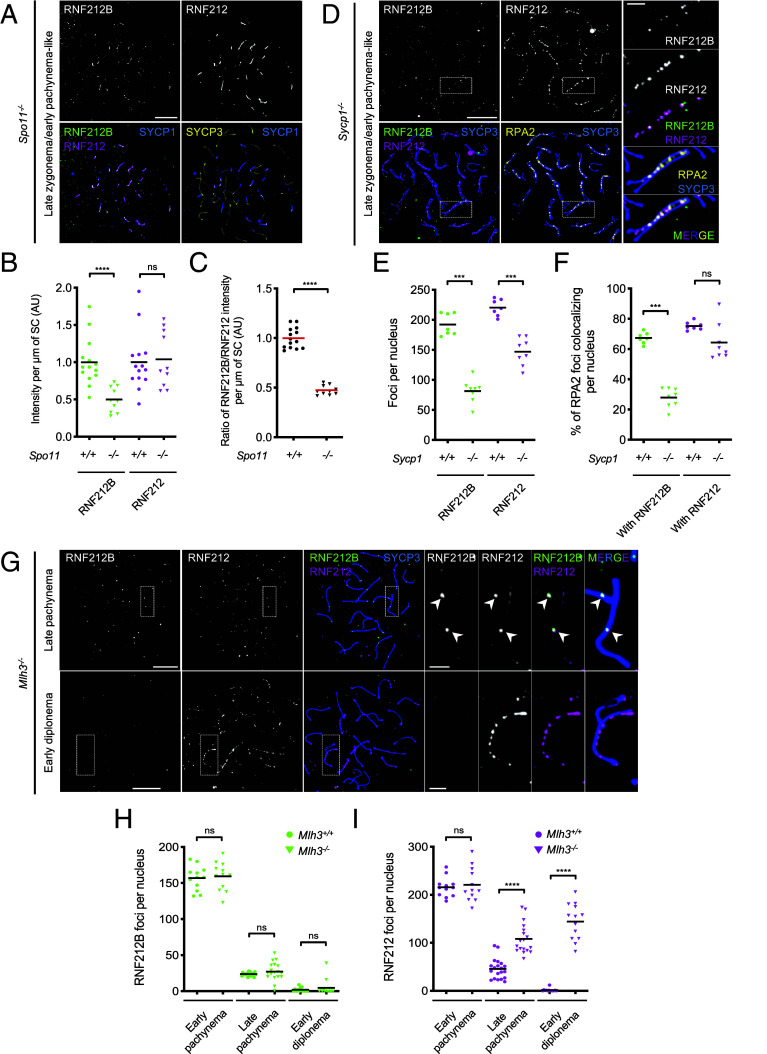
Chromosomal localization of RNF212B and RNF212 in mutants defective for recombination and synapsis (*A*) RNF212B and RNF212 localize to synapsed regions between nonhomologous axes in DSB-defective *Spo11^−/−^* spermatocytes. Images show a late zygotene/early pachytene-like nucleus immunostained for SYCP3, SYCP1, RNF212B, and RNF212. (*B* and *C*) Staining intensities of RNF212B and RNF212 on synapsed regions. Signal intensities per μm of SC per nucleus (*B*); and ratios of intensities per μm of SC for RNF212B relative to RNF212 (*C*). Black and red bars indicate means. 14 early pachytene *Spo11^+/+^* nuclei and 10 late zygotene/early pachytene-like *Spo11^−/−^* nuclei were analyzed. (*D*) RNF212B and RNF212 localize to recombination sites between aligned homolog axes in synapsis-defective *Sycp1^−/−^* spermatocytes. A late zygotene/early pachytene-like nucleus immunostained for SYCP3, RNF212B, RNF212, and RPA2 is shown. (*E* and *F*) Focus counts of RNF212B and RNF212 (*E*); and degree of RPA2 colocalization with RNF212B and RNF212 (*F*). Black bars indicate means. 7 early pachytene *Sycp1^+/+^* nuclei and 8 late zygotene/early pachytene-like *Sycp1^−/−^* nuclei were analyzed. (*G*) Differentiation of crossover-specific foci of RNF212B and RNF212 and persistence of RNF212 staining in crossover-defective *Mlh3^−/−^* spermatocyte nuclei. Late pachytene and early diplotene nuclei immunostained for SYCP3, RNF212B, and RNF212 are shown. Arrowheads highlight large RNF212B foci. (*H* and *I*) Numbers of RNF212B (*H*) and RNF212 (*I*) foci per nucleus in successive late prophase I stages of spermatocytes. Black bars indicate means. Numbers of nuclei analyzed in early pachynema, late pachynema, and early diplonema were 12, 20, and 13 for *Mlh3^+/+^*; and 12, 18, and 13 for *Mlh3^−/−^*. ns, not significant (*P* >0.05); ***P ≤0.001; ****P ≤0.0001, two-tailed Mann–Whitney tests. Magnified images in (*D*) and (*G*) show representative chromosomes. (Scale bars, 10 μm for full nuclei and 2 μm for magnified images.)

Relationships between RNF212B and RNF212 were further explored by analyzing their localization in mutants defective for recombination or synapsis. SPO11-induced DSBs are required for synapsis between homologs. However, nonhomologous synapsis can occur in *Spo11^−/−^* meiocytes (50, 51) and RNF212B specifically localized to these sites, as shown previously for RNF212 (Fig. 5*A*) (15). Intensities of RNF212B immunofluorescence in *Spo11^−/−^* nuclei with late zygotene/early pachytene-like morphologies (well-developed SYCP3-staining axes and extensive synapsis, albeit nonhomologous) were ~50% lower than equivalent early-pachytene *Spo11^+/+^* nuclei (Fig. 5*B*). By contrast, intensities of RNF212 staining were comparable for *Spo11^−/−^* and *Spo11^+/+^* nuclei such that the normalized ratio of RNF212B/RNF212 intensity was reduced by ~50% in *Spo11^−/−^* spermatocytes (Fig. 5*C*). Thus, SPO11-initiated recombination promotes association of RNF212B but not RNF212 with SCs, with only RNF212B showing a significant dependence.

SYCP1 is the major component of the SC central region. Although *Sycp1^−/−^* meiocytes fail to synapse, recombination initiates normally and homologs coalign (52). In *Sycp1^−/−^* spermatocytes, RNF212B staining was barely detectable in early/mid zygotene-like nuclei indicating that initial association is strongly dependent on the SC central region (*SI Appendix*, Fig. S12*A*). However, dim RNF212B foci formed between aligned axes in late zygotene/early pachytene-like nuclei (Fig. 5*D*); and most foci localized to recombination sites, revealed by a high level of RNF212B-RPA2 colocalization (71.0 ± 8.0%; mean ± SD, *n* = 8 nuclei), as seen previously for RNF212 (Fig. 5*D* and *SI Appendix*, Fig. S12*B*) (15).

RNF212B foci were reduced 2.4-fold in *Sycp1^−/−^* nuclei (Fig. 5*E*). Correspondingly, RPA2 colocalization with RNF212B was 2.4-fold lower (Fig. 5*F*); and RNF212B-RPA2 cofoci were reduced 1.9-fold (*SI Appendix*, Fig. S12*C*). In contrast, numbers of RNF212-RPA2 cofoci remained essentially unchanged despite a 1.5-fold reduction in total RNF212 foci (Fig. 5 *E* and *F* and *SI Appendix*, Fig. S12*C*). Consequently, colocalization of RNF212B and RNF212 foci was reduced in *Sycp1^−/−^* spermatocytes (*SI Appendix*, Fig. S12*D*).

Thus, normal localization of RNF212B to prophase-I chromosomes is strongly dependent on synapsis, with respect to both timing and abundance. Although RNF212B can independently localize to recombination sites, it shows a much greater dependence on synapsis than RNF212.

MLH3 and MLH1 constitute the MutLγ complex that facilitates maturation and crossover-specific resolution of double-Holliday junctions (29, 30). Consistently, *Mlh3^−/−^* meiocytes are proficient for synapsis and early steps of recombination, but defective for crossing over (53). We previously showed that MutLγ constrains HEI10 localization both temporally and spatially; in *Mlh3^−/−^* spermatocytes, HEI10 foci appear during zygotene, much earlier than in WT, are much more numerous (~90 per nucleus), and persist throughout pachynema (16). By contrast, RNF212B staining in *Mlh3^−/−^* spermatocytes was comparable to WT: Foci peaked in early pachynema, then diminished with 1-2 large RNF212B foci emerging on each SC (Fig. 5 *G* and *H* and *SI Appendix*, Fig. S12 *E* and *F*). Numbers of crossover-specific RNF212B foci were slightly lower *Mlh3^−/−^* nuclei than in WT, possibly reflecting reduced stability or efficiency of formation (*SI Appendix*, Fig. S12*F*). In addition, some smaller foci remained until late pachynema suggesting that an additional signal following the designation of crossover sites may trigger complete loss of RNF212B from noncrossover sites. Nonetheless, this analysis indicates that differentiation of RNF212B into crossover-specific foci precedes crossing over and does not require MutLγ. Importantly, we can also infer that while crossover-specific patterning of RNF212B requires HEI10 (above, Fig. 4 *A*–*D*), it does not require HEI10 to undergo crossover-specific patterning, i.e. crossover-specific patterning of HEI10 appears to be downstream of initial crossover designation and requires a maturation step that is dependent on MutLγ.

Initial RNF212 staining in *Mlh3^−/−^* spermatocytes was comparable to WT but differentiation of crossover-specific foci was not as conspicuous; and overall focus numbers remained relatively high throughout pachynema (Fig. 5 *G* and *I* and *SI Appendix*, Fig. S12 *E* and *G*). Numerous RNF212 foci persisted on synapsed regions into early diplotene when RNF212B staining was barely detectable. Together, localization studies in mutant contexts extend our inference that the dynamics and regulation of RNF212B, RNF212, and HEI10 are distinct.

### RNF212B Is Essential for Crossing Over in Oocytes.

*Rnf212b^−/−^* females were also sterile but unlike males, in which gametes were absent (*SI Appendix*, Fig. S3), large numbers of oocytes were made (*SI Appendix*, Fig. S13 *A* and *B*). However, analysis of MLH1 foci in prophase-I nuclei (from fetal ovaries, *SI Appendix*, Fig. S13*C*), and chiasmata in metaphase-I oocytes (from adult ovaries, *SI Appendix*, Fig. S13*D*) revealed that a severe crossover defect is the common cause of sterility in *Rnf212b^−/−^* mutants of both sexes. MLH1 foci were absent, and chiasmata were reduced >20-fold in mutant oocytes (1.11 ± 0.92 chiasmata per nucleus in *Rnf212b*^−/−^, *n* = 38 oocytes; compared to 24.6 ± 2.4 in *Rnf212b*^+/+^, *n* = 28 oocytes; means ± SDs).

### Sexually Dimorphic Localization of RNF212B, RNF212, and HEI10.

**Fig. 6.**
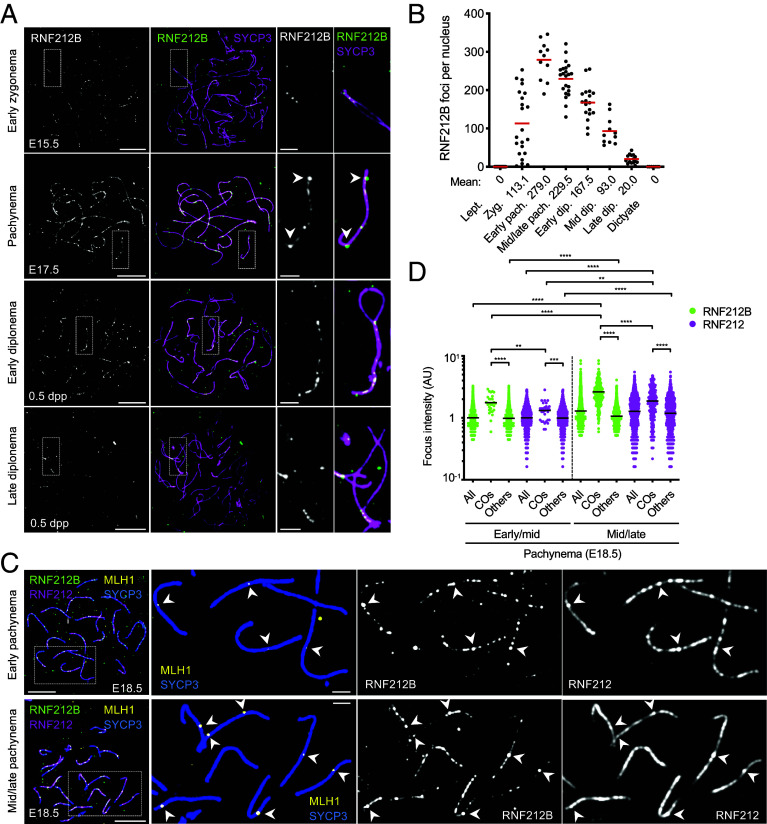
RNF212B is required for crossing over and localizes to SCs and recombination sites in prophase I oocytes. (*A*) RNF212B localization in fetal oocyte nuclei at successive prophase-I stages from embryonic days 15.5 (E15.5) and 17.5 (E17.5), and 0.5 d postpartum (0.5 dpp). Surface-spread prophase-I oocyte nuclei were immunostained for SYCP3 and RNF212B. Arrowheads indicate amplified RNF212B foci. Magnified images highlight representative chromosomes. (Scale bars, 10 μm for images of full nuclei and 2 μm for magnified panels.) (*B*) Numbers of RNF212B foci in oocyte nuclei at successive prophase-I stages. Red bars indicate means. Means of focus numbers are indicated below the graph. Numbers of nuclei analyzed in leptonema (at E15.5), zygonema (at E15.5), early pachynema (pachytene nuclei with ≤4 MLH1 foci at E16.5), mid/late pachynema (pachytene nuclei with ≥20 MLH1 foci at E18.5), early diplonema (at 0.5 dpp), mid diplonema (at 0.5 dpp), late diplonema (at 0.5 dpp), and dictyate stage (at 0.5 dpp) are 21, 22, 11, 22, 18, 11, 15, and 7, respectively. (*C*) RNF212B colocalization with RNF212 and MLH1 in oocytes. Early pachytene (*Top*) and mid/late pachytene (*Bottom*) oocytes from E18.5 ovaries immunostained for SYCP3, RNF212B, RNF212, and MLH1. Arrowheads indicate crossover sites. Magnified images show representative chromosomal regions. (Scale bars, 10 μm for images of full nuclei and 2 μm for magnified panels.) (*D*) Quantification of focus intensities for RNF212B and RNF212. Black bars indicate means. **P ≤0.01; ***P ≤0.001; ****P ≤0.0001, two-tailed Mann–Whitney tests. 7 early/mid pachytene (with ≤10 MLH1 foci) and 9 mid/late pachytene (with ≥20 MLH1 foci) nuclei from E18.5 ovaries were analyzed. Total numbers of foci analyzed: 28 crossover RNF212B foci, 1,375 other RNF212B foci, 30 crossover RNF212 foci and 1,596 other RNF212 foci in early/mid pachynema; 250 crossover RNF212B foci, 1,468 other RNF212B foci, 245 crossover RNF212 foci and 1,580 other RNF212 foci. All, all foci; COs, crossover foci colocalized with MLH1 foci; Others, other foci that do not colocalize with MLH1 foci.

RNF212B: In fetal oocytes, the initial pattern of RNF212B during zygonema was similar to spermatocytes, with foci localized to regions of synapsis (Fig. 6 *A* and *B*). Focus numbers also peaked in early pachynema (defined as pachytene oocytes with ≤4 MLH1 foci from embryonic day E16.5 ovaries) but were 1.6-fold higher than in spermatocytes (Fig. 6*B*; 279.0 ± 50.4 foci per oocyte, *n =* 11, versus 175.9 ± 15.2 foci per spermatocyte, *n =* 27; mean ± S.D.). Also distinct from spermatocytes, high numbers of RNF212B foci persisted throughout pachynema both during and after emergence of amplified foci at designated crossover sites. Indeed, even after a full complement of crossover sites had matured in mid/late pachytene oocytes (i.e. ≥20 MLH1 foci, Fig. 6*C*), RNF212B foci averaged 229.5 ± 45.0 per nucleus (from E18.5 ovaries; mean ± SD; *n =* 22; Fig. 6*B*). Furthermore, RNF212B remained on synapsed regions throughout diplonema, disappearing only as oocytes entered the dictyate stage (Fig. 6 *A* and *B*).

Given the distinct dynamics of RNF212B in oocytes, we also analyzed the timing and amplification of RNF212B and RNF212 at mature crossover sites marked by MLH1 (Fig. 6 *C* and *D*). In early/mid pachytene oocytes (≤10 MLH1 foci from E18.5 ovaries), although crossover-associated RNF212B foci were on average 1.8-fold brighter than other (noncrossover) RNF212B foci in the same nuclei, their intensity distributions overlapped (Fig. 6 *D*); indeed, we readily observed individual SCs with crossover and non-crossover RNF212B foci of similar intensities (Fig. 6 *C*, *Upper* panels).

By mid/late-pachynema (≥20 MLH1 foci from E18.5 ovaries), crossover-associated RNF212B foci were now 2.5-fold brighter than other foci, suggesting continued amplification after initial designation (Fig. 6*D*). Thus, in oocytes, growth/amplification of RNF212B may be coincident with maturation of crossover-sites marked by the emergence of MLH1 foci. This contrasts spermatocytes, in which amplified RNF212B foci clearly precede the appearance of MLH1 (Fig. 3). Again, while crossover-associated RNF212B foci were brighter than other foci at the population level, they were not always the brightest along the same SC (Fig. 6 *C*, *Lower* panels). Moreover, a consistent pattern of RNF212B foci at designated crossover sites was not observed: Some crossover foci were adjacent to other similarly bright foci, others were embedded in a domain of fainter foci, and others by gaps of diminished RNF212B staining (Fig. 6*C*). Notably, intensities of other (noncrossover) RNF212B foci and total signal intensity per oocyte nucleus were largely unchanged throughout pachynema (Fig. 6*D* and *SI Appendix*, Fig. S14*A*), in sharp contrast to the loss of RNF212B foci from non-crossover sites seen in spermatocytes (Figs. 2 and 3 and *SI Appendix*, Fig. S7).

RNF212: Abundant general RNF212 staining was also retained throughout pachynema in oocytes but amplification at crossover sites was less pronounced than RNF212B (Fig. 6*C*). Crossover-associated RNF212 foci in early/mid pachynema were only 1.3-fold brighter than other foci. By mid/late pachynema, crossover-associated RNF212 foci were now 1.6-fold brighter than other foci in the same nuclei and 1.9-fold brighter than those in early/mid pachytene consistent with continued amplification (Fig. 6*D*). Intriguingly, the intensity of RNF212 at noncrossover sites slightly increased in mid/late pachynema nuclei suggesting general loading may continue (Fig. 6*D*). Moreover, total signal intensity per oocyte nucleus was largely unchanged between early/mid and mid/late pachynema, as seen for RNF212B (*SI Appendix*, Fig. S14*A*).

**Fig. 7.**
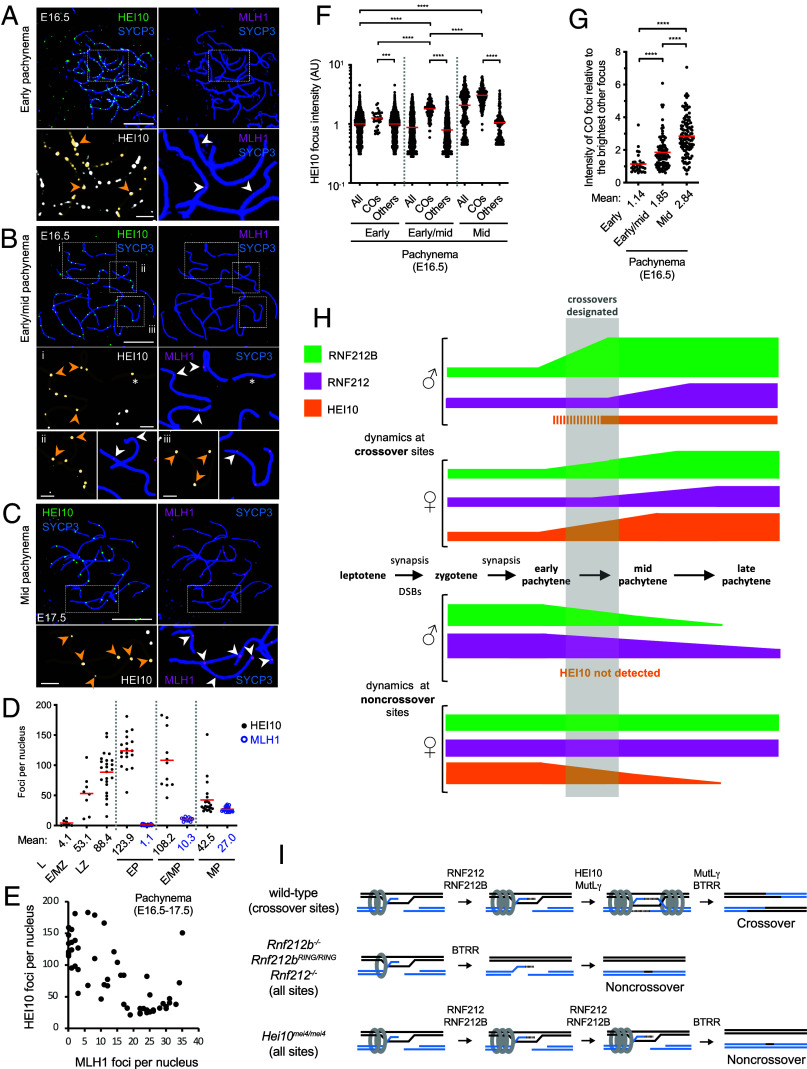
Dynamic chromosomal localization of HEI10 in oocytes. (*A*–*C*) HEI10 localization at successive prophase-I stages of fetal oocyte nuclei from embryonic days 16.5 (E16.5) and 17.5 (E17.5). Prophase-I oocyte nuclei in early pachynema (*A*), early/mid pachynema (*B*), and mid pachynema (*C*) were immunostained for SYCP3, HEI10, and MLH1. Arrowheads indicate crossover foci marked by MLH1. An asterisk indicates a differentiated/bright HEI10 focus that lacks MLH1 focus on a chromosome. Magnified images show representative chromosomal regions. (Scale bars, 10 μm for full nuclei and 2 μm for magnified panels.) (*D*) Numbers of HEI10 and MLH1 foci in oocyte nuclei at successive prophase-I stages. Red bars indicate means. Means of focus numbers are indicated below the graph. Numbers of nuclei analyzed in leptonema (L; at E16.5), early and mid zygonema (E/MZ; at E16.5), late zygonema (LZ; at E16.5), early pachynema (pachytene nuclei with ≤4 MLH1 foci at E16.5), early-to-mid pachynema (E/MP; pachytene nuclei with 5-15 MLH1 foci at E16.5), and mid pachynema (MP; pachytene nuclei with ≥20 MLH1 foci at E16.5 and E17.5), are 8, 8, 24, 18, 11, and 21, respectively. (*E*) Quantification of HEI10 and MLH1 foci in pachytene oocytes from E16.5 and E17.5 ovaries. 56 pachytene nuclei were randomly selected and analyzed. (*F*) Quantification of HEI10 focus intensities. Red bars indicate means. ***P ≤0.001; ****P ≤0.0001, two-tailed Mann–Whitney tests. 26 early pachytene (with ≤4 MLH1 foci), 9 early/mid pachytene (with 5-15 MLH1 foci), and 6 mid pachytene (with ≥20 MLH1 foci) nuclei from E16.5 ovaries were analyzed. Total numbers of foci analyzed: 33 crossover foci and 3,817 other foci in early pachynema; 96 crossover foci and 1,089 other foci in early/mid pachynema; 186 crossover foci and 180 other foci. All, all foci; COs, crossover foci colocalized with MLH1 foci; Others, foci that do not colocalize with MLH1 foci. (*G*) Per chromosome analysis for HEI10 focus intensity. Intensity of each crossover-associated HEI10 focus relative to that of the brightest other (non-crossover) focus along the same chromosome. Red bars indicate means. Mean intensities are indicated below the graph. Detailed representation is shown in *SI Appendix*, Fig. S14*C*. ****P ≤0.0001, two-tailed Mann–Whitney tests. 31, 92, and 111 crossover foci from 14 early pachytene, 9 early/mid pachytene, and 6 mid pachytene nuclei were analyzed, respectively. (*H*) Dynamics of CORs relative to substages of meiotic prophase-I in spermatocytes and oocytes. Accumulation of RNF212B at prospective crossover sites is stronger and occurs earlier than RNF212, coincident with the appearance of HEI10 foci in spermatocytes and amplification of HEI10 in oocytes. RNF212B is lost from noncrossover sites earlier than RNF212 in spermatocytes, while noncrossover foci of both RNF212B and RNF212 persist throughout pachynema in oocytes. In oocytes, HEI10 foci are present at noncrossover sites at the time of crossover designation but eventually diminish. (*I*) Models of crossover maturation and defects in COR mutants. MutSγ complexes (gray rings) bind Holliday junctions and convert into sliding clamps that embrace interacting duplexes (28). At designated crossover sites, stabilization of MutSγ by CORs enables formation of double-Holliday junctions. An endonuclease-independent function of MutLγ is recently implicated in this step (59). MutLγ-catalyzed incision and the Bloom complex (BLM-TOPIIIα-RMI1-RMI2; BTRR) then mediate crossover-specific resolution (29). MutSγ is destabilized in *Rnf212b^−/−^, Rnf212b^RING/RING^*, and *Rnf212^−/−^* mutants exposing nascent intermediates to dissociation by the Bloom complex resulting in noncrossovers. In the *Hei10^mei4/mei4^* mutant, RNF212, RNF212B, and MutSγ persist at all sites throughout pachytene, and crossovers fail to differentiate. DSBs are repaired as noncrossovers only after homologs desynapse.

HEI10: Localization of HEI10 was also sexually dimorphic. Unlike spermatocytes, prominent HEI10 foci were already apparent in zygotene oocytes, specifically at regions of synapsis (*SI Appendix*, Fig. S14*B*). Focus numbers increased as synapsis ensued, peaking in early pachynema (oocytes with ≤4 MLH1 foci) at 123.9 ± 28.7 foci per nucleus (mean ± SD; *n =* 18; Fig. 7 *A* and *D*). At this stage, HEI10 foci were unevenly spaced and highly variable in size and intensity; with configurations suggestive of growth, shrinkage, and/or fusion as might be predicted by coarsening models (Fig. 7 *A* and *F* and *SI Appendix*, Fig. S14 *B* and *D*). Focus numbers then reduced, developing into a crossover-specific pattern by mid pachynema (oocytes with ≥20 MLH1 foci from E17.5 ovaries; Fig. 7 *B*–*E*). At this stage, 86.2 ± 9.6% of HEI10 foci colocalized with MLH1 and 98.0 ± 2.7% of MLH1 foci colocalized with HEI10 (means ± SDs; *n =* 14).

Crossover-associated HEI10 foci in early-mid pachytene oocytes (5-15 MLH1 foci from E16.5 ovaries) were on average 2.3-fold brighter than other foci (Fig. 7 *B* and *F*). Even very faint emergent MLH1 foci in early pachytene oocytes (with ≤4 MLH1 foci from E16.5 ovaries) tended to be associated with slightly larger HEI10 foci suggesting that amplification accompanies crossover designation (Fig. 7 *A* and *F*). However, MLH1 foci emerged when HEI10 foci still outnumbered crossovers by more than 3:1, i.e. before HEI10 had attained a crossover-specific distribution. Moreover, while crossover-associated HEI10 foci in early-mid pachynema were on average 1.9-fold brighter than the brightest other focus along the same chromosome (Fig. 7*G*), similarly bright HEI10 foci were often observed along the same SC (Fig. 7*B*, magnified panels, and *SI Appendix*, Fig. S14 *B* and *C*). After mid pachynema (≥20 MLH1 foci from E16.5 ovaries), crossover-associated HEI10 foci were 3.1-fold brighter than the foci detected in early pachynema and 1.7-fold brighter than the crossover-specific foci detected in early-mid pachynema (Fig. 7*F*), suggesting continued amplification of HEI10 after crossover designation.

Together, these data reveal profound sexual dimorphism in the localization dynamics of RNF212B, RNF212, and HEI10. Importantly, in oocytes, designation of crossover sites does not involve general depletion of RNF212B and RNF212 from noncrossover sites; and amplification may be coincident or downstream. Distinctly, HEI10 amplifies at designated crossover sites while progressively diminishing from other sites. While HEI10 amplification could play a role in crossover-site designation, crossover sites are maturing well before a crossover-specific pattern of HEI10 is attained.

## Discussion

### COR Patterning Vis-a-Vis Crossover Patterning.

An elementary coarsening model, that can reproduce crossover patterning in Arabidopsis, posits that a finite amount of HEI10 (fixed amount per unit length) diffuses along SCs and becomes progressively absorbed into one or a few amplified foci to designate which recombination sites will mature into crossovers (35, 36). Our data in the mouse are hard to reconcile with this model, revealing a more complex scenario in which patterning of a given COR protein may precede, accompany, or follow apparent designation of crossover sites; can occur with or without depletion of CORs from non-crossover sites; and shows striking sexual dimorphism (summarized in Fig. 7*H*). A challenge for the future is to reconcile these data with an alternative model of crossover patterning.

### Global and Local Regulation of Recombination by RNF212B, RNF212, and HEI10.

What might be the function(s) of the observed dynamics of mammalian CORs? Our analysis emphasizes interdependencies and distinctions between RNF212B, RNF212, and HEI10 and their global and local functions that help coordinate key events of meiotic prophase. Most evident is regulating the progression of recombination in the context of the SC. Following initial DNA strand-exchange and homolog pairing, RNF212-RNF212B associates with nascent SCs and acts to stabilize ZMM factors and pause the progression of recombination. In this way, RNF212-RNF212B may protect nascent recombination intermediates from premature dissociation by the Bloom complex, which is inferred to mediate default noncrossover repair via synthesis-dependent strand annealing (31–34, 54). This early function of RNF212-RNF212B appears to stabilize synapsis, possibly acting as a proofreading mechanism to selectively reinforce SC assembled at recombination sites, i.e. between homologs. As prophase progresses, connection of homologs by recombinational interactions is superseded by the SC. RNF212-RNF212B could mediate this hand-off by ensuring recombinational connections are not resolved until mature SC forms.

Pausing SC-associated recombination may also be a prerequisite for crossover/noncrossover differentiation and/or to allow time for crossover sites to mature. Importantly, RNF212-RNF212B renders progression dependent on HEI10 (Fig. 4) and additional pro-crossover factors including kinase CDK2, and the CNTD1–PRR19 complex (16, 17, 46, 55, 56). In the absence of HEI10, RNF212-RNF212B and ZMM foci remain abundant and undifferentiated along SCs, and recombination remains stalled, with DSB repair being completed only as the SC is disassembled (Fig. 7*I*).

At designated crossover sites, continued protection of intermediates via RNF212-RNF212B may enable crossover-specific events including dHJ formation and recruitment of crossover-specific resolution machinery, organized around the MutLγ endonuclease (Fig. 7*I*). Local stabilization of SC via RNF212-RNF212B may also explain why crossover sites are the last sites to desynapse during diplotene (57). We have suggested that patches of SC retained at crossover sites help coordinate DNA events of crossing over with exchange of underlying chromosome axes to form chiasmata.

### Distinct and Interdependent Functions of RNF212, RNF212B, and HEI10.

Our ability to discern distinct functions of RNF212 and RNF212B is compromised by their interdependence for protein stability, i.e., *Rnf212* and *Rnf212b* single mutant phenotypes likely reflect diminished function of both proteins. However, localization dynamics of RNF212 and RNF212B reveal divergent behaviors that point to distinct functions and regulation.

Notably, initial RNF212 foci outnumber RNF212B and up to 40% do not detectably colocalize. Also, amplification of crossover-specific RNF212B foci is much stronger than RNF212 and occurs earlier, with the suggestion that differentiation may occur via distinct processes, e.g. redistribution to crossover sites for RNF212B, versus loss from noncrossover sites for RNF212 (at least via different proportions of redistribution and loss). Thus, although RNF212 and RNF212B interact, these observations argue against an obligate heterocomplex of fixed stoichiometry and suggest RNF212B function may be modulated by switching binding partners as it accumulates at designated crossover sites. For example, RNF212B–RNF212B self-interaction could become prominent, or RNF212B could partner with HEI10 to locally accumulate and modulate E3 ligase activities at designated crossover sites.

Localization in mutant backgrounds also points to distinct functions and regulation of the three CORs. RNF212 localization along SCs remains robust without SPO11-catalyzed DSBs while RNF212B localizes less efficiently, suggesting DSB signaling stabilizes RNF212B. In this respect, RNF212B may be like budding yeast Zip3, which requires phosphorylation by DNA-damage response kinases Mec1^ATR^/Tel1^ATM^ for normal localization (58). Further distinctions are seen in the absence of synapsis, with RNF212 robustly localizing to recombination sites, while RNF212B is much weaker. Thus, inputs from both DSBs and synapsis stabilize RNF212B and thereby coordinate its activity in space and time, while RNF212 might act more as an anchor for localization to SCs and recombination sites. HEI10 foci in spermatocytes are largely dependent on both DSBs and synapsis (16), consistent with our inference that they assemble only as crossover sites differentiate, dependent on RNF212-RNF212B.

Defective crossover maturation in the *Mlh3* mutant impacts COR dynamics to varying degrees. Although crossover-specific patterning of RNF212-RNF212B foci still occurs in *Mlh3*^−/−^ spermatocytes, numerous small foci of RNF212, and to a lesser extent RNF212B, persist implying that a MutLγ-dependent signal associated with maturation of crossover sites enhances general loss from noncrossover sites. HEI10 patterning is severely perturbed in *Mlh3*^−/−^ spermatocytes, with high numbers of foci forming much earlier than normal (in zygonema) and then persisting until chromosomes desynapse (16). Thus, although HEI10 is required for crossover patterning of RNF212-RNF212B foci, this function does not require crossover-specific patterning of HEI10, i.e. crossover-specific localization of HEI10 appears to be downstream of initial crossover designation. Furthermore, crossover-specific patterning of HEI10 requires an upstream maturation step that is dependent on the MutLγ complex, likely the dHJ formation function described by Premkumar et al. (59). Consistently, in oocytes, MLH1 foci can be detected before HEI10 foci have attained a crossover-specific pattern (Fig. 7*A* and *SI Appendix*, Fig. S14 *B* and *C*).

Collectively, these data suggest that CORs function as apical effectors that coordinate meiotic prophase by integrating signals from synapsis, DSB repair, cell cycle kinases, and maturating crossover sites (summarized in Fig. 7*I*).

### Sexual Dimorphism.

Patterning of all three CORs shows striking sexual dimorphism. In spermatocytes, complete differentiation of RNF212B results in only large crossover-specific foci; while in oocytes, numerous foci persist throughout pachytene even after amplified crossover-specific foci form and crossover sites mature (Figs. 2 *A* and *B* and 6 *A* and *B*). RNF212 dynamics are similarly dimorphic. Thus, complete differentiation of RNF212B or RNF212 is not a prerequisite for the patterning and maturation of crossover sites. Possibly, accumulating a threshold level of RNF212B at a given recombination site is sufficient for it to mature into a crossover. However, weaker amplification and incomplete differentiation in females could render RNF212B crossover foci less stable and potentially reversible, possibly accounting for the inefficiency of crossover maturation characterized in human oocytes that can result in unconnected (achiasmate) homologs, or homologs connected only by a single telomere-proximal chiasma, configurations that are prone to segregation error (8, 9). HEI10 also shows striking temporal and spatial dimorphism: In spermatocytes, foci emerging in mid pachytene already show a crossover distribution; while in oocytes, numerous foci first form during zygotene and then attain a crossover-specific pattern in pachytene.

What could account for the sexually dimorphic behavior of mammalian CORs? A pertinent difference may be the duration of pachytene, which lasts almost 7 d in mouse spermatocytes (60) compared to just 1 to 2 d in oocytes (61). Thus, if patterning dynamics are relatively slow, differentiation may remain incomplete in oocytes. Also, oocytes have longer SCs and CORs appear to be generally more abundant. COR dimorphism might also reflect differences in the DNA-damage checkpoint and gamete quality control. We previously showed that RNF212 is required for quality control as oocytes arrest in dictyate and assemble into primordial follicles (62), while the primary checkpoint in spermatocytes occurs much earlier, in mid pachytene (63).

## Materials and Methods

*Rnf212b* mutant mice were generated by the Cornell Stem Cell & Transgenic Core. Mice were maintained and euthanized according to IACUC guidelines of UC Davis. Surface-spread chromosomes from prophase I and metaphase I were prepared as described (64, 65) with minor modifications (*SI Appendix*), Histology, immunostaining, immunoblotting, Y2H, antibody production, and RNA extraction were performed with standard protocols (*SI Appendix*). Details of image acquisition, processing, and analysis are described in *SI Appendix*. Statistics were performed using Graphpad Prism v.8-9 and R version 3.5.2; parameters and tests, sample sizes, means, and error bars, are described in the text, figures, or corresponding legends. Sample sizes were not predetermined using statistical tests.

## Supplementary Material

Appendix 01 (PDF)

## Data Availability

Raw images data have been deposited in Mendeley (https://doi.org/10.17632/ysnftfyd8p.1) (66).

## References

[1] Y. Watanabe, Geometry and force behind kinetochore orientation: Lessons from meiosis. Nat. Rev. Mol. Cell Biol. **13**, 370–382 (2012). 22588367 10.1038/nrm3349

[2] N. Hunter, Meiotic recombination: The essence of heredity. Cold Spring Harbor Perspect. Biol. **7**, a016618 (2015). 10.1101/cshperspect.a016618 PMC4665078 26511629

[3] D. Zickler, N. Kleckner, Meiosis: Dances between homologs. Annu. Rev. Genet. **57**, 1–63 (2023), 10.1146/annurev-genet-061323-044915. 37788458

[4] I. Faisal, L. Kauppi, Reduced MAD2 levels dampen the apoptotic response to non-exchange sex chromosomes and lead to sperm aneuploidy. Development **144**, 1988–1996 (2017). 28506992 10.1242/dev.149492

[5] T. Hassold, H. Hall, P. Hunt, The origin of human aneuploidy: Where we have been, where we are going. Hum. mol. genet. **16**, R203–R208 (2007). 17911163 10.1093/hmg/ddm243

[6] T. Hassold, P. Hunt, To err (meiotically) is human: The genesis of human aneuploidy. Nat. Revi. Genet. **2**, 280–291 (2001). 10.1038/35066065 11283700

[7] I. Faisal, L. Kauppi, Sex chromosome recombination failure, apoptosis, and fertility in male mice. Chromosoma **125**, 227–235 (2016). 26440410 10.1007/s00412-015-0542-9

[8] T. Hassold , Failure to recombine is a common feature of human oogenesis. Am. J. Hum. Genet. **108**, 16–24 (2021). 33306948 10.1016/j.ajhg.2020.11.010 PMC7820622

[9] S. Wang , Inefficient crossover maturation underlies elevated aneuploidy in human female meiosis. Cell **168**, 977–989.e917 (2017). 28262352 10.1016/j.cell.2017.02.002 PMC5408880

[10] L. Wartosch , Origins and mechanisms leading to aneuploidy in human eggs. Prenat. Diagn. **41**, 620–630 (2021). 33860956 10.1002/pd.5927 PMC8237340

[11] F. Cole , Homeostatic control of recombination is implemented progressively in mouse meiosis. Nat. Cell Biol. **14**, 424–430 (2012). 22388890 10.1038/ncb2451 PMC3319518

[12] S. Wang, D. Zickler, N. Kleckner, L. Zhang, Meiotic crossover patterns: Obligatory crossover, interference and homeostasis in a single process. Cell Cycle **14**, 305–314 (2015). 25590558 10.4161/15384101.2014.991185 PMC4353236

[13] L. Chelysheva , The Arabidopsis HEI10 is a new ZMM protein related to Zip3. PLoS Genet. **8**, e1002799 (2012). 22844245 10.1371/journal.pgen.1002799 PMC3405992

[14] C. H. Cheng , SUMO modifications control assembly of synaptonemal complex and polycomplex in meiosis of Saccharomyces cerevisiae. Genes Develop. **20**, 2067–2081 (2006). 16847351 10.1101/gad.1430406 PMC1536058

[15] A. Reynolds , RNF212 is a dosage-sensitive regulator of crossing-over during mammalian meiosis. Nat. Genet. **45**, 269–278 (2013). 23396135 10.1038/ng.2541 PMC4245152

[16] H. Qiao , Antagonistic roles of ubiquitin ligase HEI10 and SUMO ligase RNF212 regulate meiotic recombination. Nat. Genet. **46**, 194–199 (2014). 24390283 10.1038/ng.2858 PMC4356240

[17] H. B. Rao , A SUMO-ubiquitin relay recruits proteasomes to chromosome axes to regulate meiotic recombination. Science **355**, 403–407 (2017). 28059716 10.1126/science.aaf6407 PMC5569317

[18] A. De Muyt , E3 ligase Hei10: A multifaceted structure-based signaling molecule with roles within and beyond meiosis. Genes Develop. **28**, 1111–1123 (2014). 24831702 10.1101/gad.240408.114 PMC4035539

[19] L. Zhang, S. Kohler, R. Rillo-Bohn, A. F. Dernburg, A compartmentalized signaling network mediates crossover control in meiosis. eLife **7**, e30789 (2018), 10.7554/eLife.30789. 29521627 PMC5906097

[20] H. Nguyen , elegans ZHP-4 is required at multiple distinct steps in the formation of crossovers and their transition to segregation competent chiasmata. PLoS Genet. **14**, e1007776 (2018). 30379819 10.1371/journal.pgen.1007776 PMC6239344

[21] C. M. Lake , Narya, a RING finger domain-containing protein, is required for meiotic DNA double-strand break formation and crossover maturation in Drosophila melanogaster. PLoS Genet. **15**, e1007886 (2019). 30615609 10.1371/journal.pgen.1007886 PMC6336347

[22] K. Wang , The role of rice HEI10 in the formation of meiotic crossovers. PLoS Genet. **8**, e1002809 (2012). 22792078 10.1371/journal.pgen.1002809 PMC3390396

[23] C. M. Lake , Vilya, a component of the recombination nodule, is required for meiotic double-strand break formation in *Drosophila*. eLife **4**, e08287 (2015). 26452093 10.7554/eLife.08287 PMC4703084

[24] N. Bhalla, D. J. Wynne, V. Jantsch, A. F. Dernburg, ZHP-3 acts at crossovers to couple meiotic recombination with synaptonemal complex disassembly and bivalent formation in *C. elegans*. PLoS Genet. **4**, e1000235 (2008). 18949042 10.1371/journal.pgen.1000235 PMC2567099

[25] J. O. Ward , Mutation in mouse hei10, an e3 ubiquitin ligase, disrupts meiotic crossing over. PLoS Genet. **3**, e139 (2007). 17784788 10.1371/journal.pgen.0030139 PMC1959360

[26] M. Shinohara, S. D. Oh, N. Hunter, A. Shinohara, Crossover assurance and crossover interference are distinctly regulated by the ZMM proteins during yeast meiosis. Nat. Genet. **40**, 299–309 (2008). 18297071 10.1038/ng.83

[27] A. Pyatnitskaya, V. Borde, A. De Muyt, Crossing and zipping: Molecular duties of the ZMM proteins in meiosis. Chromosoma **128**, 181–198 (2019). 31236671 10.1007/s00412-019-00714-8

[28] T. Snowden, S. Acharya, C. Butz, M. Berardini, R. Fishel, hMSH4-hMSH5 recognizes Holliday Junctions and forms a meiosis-specific sliding clamp that embraces homologous chromosomes. Mol. cell **15**, 437–451 (2004). 15304223 10.1016/j.molcel.2004.06.040

[29] D. S. Kulkarni , PCNA activates the MutLgamma endonuclease to promote meiotic crossing over. Nature **586**, 623-627 (2020), 10.1038/s41586-020-2645-6. 32814343 PMC8284803

[30] E. Cannavo , Regulation of the MLH1-MLH3 endonuclease in meiosis. Nature **586**, 618-622 (2020), 10.1038/s41586-020-2592-2. 32814904

[31] L. Jessop, B. Rockmill, G. S. Roeder, M. Lichten, Meiotic chromosome synapsis-promoting proteins antagonize the anti-crossover activity of Sgs1. PLoS Genet. **2**, e155 (2006). 17002499 10.1371/journal.pgen.0020155 PMC1570379

[32] A. De Muyt , BLM helicase ortholog Sgs1 is a central regulator of meiotic recombination intermediate metabolism. Mol. Cell **46**, 43–53 (2012). 22500736 10.1016/j.molcel.2012.02.020 PMC3328772

[33] K. Zakharyevich, S. Tang, Y. Ma, N. Hunter, Delineation of joint molecule resolution pathways in meiosis identifies a crossover-specific resolvase. Cell **149**, 334–347 (2012). 22500800 10.1016/j.cell.2012.03.023 PMC3377385

[34] S. Tang, M. K. Y. Wu, R. Zhang, N. Hunter, Pervasive and essential roles of the Top3-Rmi1 decatenase orchestrate recombination and facilitate chromosome segregation in meiosis. Mol. Cell **57**, 607–621 (2015). 25699709 10.1016/j.molcel.2015.01.021 PMC4791043

[35] C. Morgan , Diffusion-mediated HEI10 coarsening can explain meiotic crossover positioning in Arabidopsis. Nat. Commun. **12**, 4674 (2021). 34344879 10.1038/s41467-021-24827-w PMC8333306

[36] J. A. Fozard, C. Morgan, M. Howard, Coarsening dynamics can explain meiotic crossover patterning in both the presence and absence of the synaptonemal complex. eLife **12**, e79408 (2023). 36847348 10.7554/eLife.79408 PMC10036115

[37] L. Zhang, W. Stauffer, D. Zwicker, D. A.F., Crossover patterning through kinase-regulated condensation and coarsening of recombination nodules. bioRxiv [Preprint] (2021). 10.1101/2021.08.26.457865 (Accessed 27 August 2021).

[38] N. Kleckner , A mechanical basis for chromosome function. Proc. Natl. Acad. Sci. U.S.A. **101**, 12592–12597 (2004). 15299144 10.1073/pnas.0402724101 PMC515102

[39] L. Zhang, Z. Liang, J. Hutchinson, N. Kleckner, Crossover patterning by the beam-film model: Analysis and implications. PLoS Genet. **10**, e1004042 (2014). 24497834 10.1371/journal.pgen.1004042 PMC3907302

[40] S. E. Johnston, M. A. Stoffel, J. M. Pemberton, Variants at RNF212 and RNF212B are associated with recombination rate variation in Soay sheep (Ovis aries). bioRxiv [Preprint] (2020). 10.1101/2020.07.26.217802 (Accessed 26 July 2020).

[41] M. Gershoni , A pathogenic variant in the uncharacterized RNF212B gene results in severe aneuploidy male infertility and repeated IVF failure. HGG Adv. **4**, 100189 (2023). 37124137 10.1016/j.xhgg.2023.100189 PMC10133878

[42] N. K. Kadri , Coding and noncoding variants in HFM1, MLH3, MSH4, MSH5, RNF212, and RNF212B affect recombination rate in cattle. Genome Res. **26**, 1323–1332 (2016). 27516620 10.1101/gr.204214.116 PMC5052053

[43] S. E. Johnston, J. Huisman, J. M. Pemberton, A genomic region containing REC8 and RNF212B is associated with individual recombination rate variation in a wild population of red deer (Cervus elaphus). G3 **8**, 2265–2276 (2018). 29764960 10.1534/g3.118.200063 PMC6027875

[44] S. E. Johnston, C. Berenos, J. Slate, J. M. Pemberton, Conserved Genetic architecture underlying individual recombination rate variation in a wild population of soay sheep (Ovis aries). Genetics **203**, 583–598 (2016). 27029733 10.1534/genetics.115.185553 PMC4858801

[45] Y. B. Condezo , RNF212B E3 ligase is essential for crossover designation and maturation during male and female meiosis in the mouse. Proc. Natl. Acad. Sci. U.S.A. **121**, e2320995121 (2024). 38865271 10.1073/pnas.2320995121 PMC11194559

[46] A. Bondarieva , Proline-rich protein PRR19 functions with cyclin-like CNTD1 to promote meiotic crossing over in mouse. Nat. Commun. **11**, 3101 (2020). 32555348 10.1038/s41467-020-16885-3 PMC7303132

[47] T. Ashley, D. Walpita, D. G. de Rooij, Localization of two mammalian cyclin dependent kinases during mammalian meiosis. J. Cell Sci. **114**, 685–693 (2001). 11171374 10.1242/jcs.114.4.685

[48] M. S. Brown, D. K. Bishop, DNA strand exchange and RecA homologs in meiosis. Cold Spring Harbor Perspect. Biol. **7**, a016659 (2014). 10.1101/cshperspect.a016659 PMC4292170 25475089

[49] A. G. Hinch , The configuration of RPA, RAD51, and DMC1 binding in meiosis reveals the nature of critical recombination intermediates. Mol. Cell **79**, 689–701.e610 (2020). 32610038 10.1016/j.molcel.2020.06.015 PMC7447979

[50] F. Baudat, K. Manova, J. P. Yuen, M. Jasin, S. Keeney, Chromosome synapsis defects and sexually dimorphic meiotic progression in mice lacking Spo11. Mol. Cell **6**, 989–998 (2000). 11106739 10.1016/s1097-2765(00)00098-8

[51] P. J. Romanienko, R. D. Camerini-Otero, The mouse Spo11 gene is required for meiotic chromosome synapsis. Mol. Cell **6**, 975–987 (2000). 10.1016/s1097-2765(00)00097-6 11106738

[52] F. A. de Vries , Mouse Sycp1 functions in synaptonemal complex assembly, meiotic recombination, and XY body formation. Genes Develop. **19**, 1376–1389 (2005). 15937223 10.1101/gad.329705 PMC1142560

[53] S. M. Lipkin , Meiotic arrest and aneuploidy in MLH3-deficient mice. Nat. Genet. **31**, 385–390 (2002). 12091911 10.1038/ng931

[54] M. S. McMahill, C. W. Sham, D. K. Bishop, Synthesis-dependent strand annealing in meiosis. PLoS Biol. **5**, e299 (2007). 17988174 10.1371/journal.pbio.0050299 PMC2062477

[55] N. Palmer , A novel function for CDK2 activity at meiotic crossover sites. PLoS Biol. **18**, e3000903 (2020). 33075054 10.1371/journal.pbio.3000903 PMC7595640

[56] J. K. Holloway, X. Sun, R. Yokoo, A. M. Villeneuve, P. E. Cohen, Mammalian CNTD1 is critical for meiotic crossover maturation and deselection of excess precrossover sites. J. Cell Biol. **205**, 633–641 (2014). 24891606 10.1083/jcb.201401122 PMC4050721

[57] H. Qiao , Interplay between synaptonemal complex, homologous recombination, and centromeres during mammalian meiosis. PLoS Genet. **8**, e1002790 (2012). 22761591 10.1371/journal.pgen.1002790 PMC3386176

[58] M. E. Serrentino, E. Chaplais, V. Sommermeyer, V. Borde, Differential association of the conserved SUMO ligase Zip3 with meiotic double-strand break sites reveals regional variations in the outcome of meiotic recombination. PLoS Genet. **9**, e1003416 (2013). 23593021 10.1371/journal.pgen.1003416 PMC3616913

[59] T. Premkumar , Genetic dissection of crossover mutants defines discrete intermediates in mouse meiosis. Mol. Cell **83**, 2941–2958 (2023). 37595556 10.1016/j.molcel.2023.07.022 PMC10469168

[60] G. W. van der Heijden , Chromosome-wide nucleosome replacement and H3.3 incorporation during mammalian meiotic sex chromosome inactivation. Nat. Genet. **39**, 251–258 (2007). 17237782 10.1038/ng1949

[61] F. Ghafari, C. G. Gutierrez, G. M. Hartshorne, Apoptosis in mouse fetal and neonatal oocytes during meiotic prophase one. BMC Dev. Biol. **7**, 87 (2007). 17650311 10.1186/1471-213X-7-87 PMC1965470

[62] H. Qiao , Impeding DNA break repair enables oocyte quality control. Mol. Cell **72**, 211–221.e213 (2018). 30270110 10.1016/j.molcel.2018.08.031 PMC6426715

[63] H. Abe , The initiation of meiotic sex chromosome inactivation sequesters DNA damage signaling from autosomes in mouse spermatogenesis. Curr. Biol. **30**, 408–420.e405 (2020). 31902729 10.1016/j.cub.2019.11.064 PMC7076562

[64] M. Ito , FIGNL1 AAA+ ATPase remodels RAD51 and DMC1 filaments in pre-meiotic DNA replication and meiotic recombination. Nat. Commun. **14**, 6857 (2023). 37891173 10.1038/s41467-023-42576-w PMC10611733

[65] Y. Yun, M. Ito, S. Sandhu, N. Hunter, Cytological monitoring of meiotic crossovers in spermatocytes and oocytes. Methods Mol. Biol. **2153**, 267–286 (2021). 32840786 10.1007/978-1-0716-0644-5_19

[66] M. Ito, Distinct and interdependent functions of three RING proteins regulate recombination during mammalian meiosis. Ito et al. Mendeley Data, V1. doi: 10.17632/ysnftfyd8p.1. Deposited 18 November 2024. PMC11745341 39761402

