# Supplementary material for: Distinct and interdependent functions of three RING proteins regulate recombination during mammalian meiosis
Source: Proc Natl Acad Sci U S A. 2025 Jan 6;122(2):e2412961121. doi: 10.1073/pnas.2412961121 (PMC11745341; doi:10.1073/pnas.2412961121)
Supplement: Supplementary file 1 — Appendix 01 (PDF) [file pnas.2412961121.sapp.pdf]

PNAS

Supporting Information for

Distinct and interdependent functions of three RING proteins regulate recombination during mammalian meiosis.

Masaru Ito[1,2,5,*], Yan Yun[1,2,6], Dhananjaya S. Kulkarni[1,2], Sunkyung Lee[1,2], Sumit Sandhu[1,2], Briana Nuñez[1,3], Linya Hu[2], Kevin Lee[2], Nelly Lim[2], Rachel M. Hirota[2], Rowan Prendergast[2], Cynthia Huang[2], Ivy Huang[2] and Neil Hunter[1,2,4,5 *]
[1] Howard Hughes Medical Institute, University of California Davis, Davis, CA 95616, USA
[2] Department of Microbiology & Molecular Genetics, University of California Davis, Davis, CA 95616, USA
[3] Department of Biochemistry & Molecular Biology, Brown University, Providence, RI 02912, USA
[4] Department of Molecular & Cellular Biology, University of California Davis, Davis, CA 95616, USA
[5] Institute for Protein Research, Osaka University, Osaka 565-0871, Japan
[6] Center for Reproductive Medicine, Clinical Research Center, Shantou Central Hospital, Shantou, China 515041
*Correspondence: Neil Hunter and Masaru Ito
Email: nhunter@ucdavis.edu, msrito2@protein.osaka-u.ac.jp

This PDF file includes:

Supporting text
Figures S1 to S14
Tables S1 to S3
SI References

Supporting Information Text

Materials and Methods

Mice

All mice were congenic with the C57BL/6J background except for *Sycp1*$^{-/-}$ mutant mice. The *Rnf212*$^{-/-}$, *Hei10*$^{mei4/mei4}$, *Spo11*$^{-/-}$, *Sycp1*$^{-/-}$ and *Mlh3*$^{-/-}$ mutant lines were previously described (1-5). Mature adult mice (2-6 months old) were used for experiments unless otherwise noted.

For generation of *Rnf212b* mutant alleles, 124 bp of DNA template for sgRNA, comprising the T7 polymerase binding site and sgRNA target sequence (5'-CCCCATCTTTTCGGAAACAC-3') followed by the remaining sgRNA sequence, were amplified by PCR as previously described (6) and purified using a GeneJET PCR Purification Kit (Thermo Scientific, K0702). 600 ng of purified DNA template was *in vitro* transcribed for 4 h at 37°C using MEGAshortscript T7 Transcription Kit (Invitrogen, AM1354). sgRNA was purified using MEGAclear Transcription Clean-Up Kit (Invitrogen, AM1908), diluted to 1.6 µg/µl and stored at -80°C prior to embryo injection. 12.5 ng/µl of purified sgRNA and either 12.5 or 25 ng/µl of Cas9 mRNA were microinjected into pronuclei of 2-cell stage of C57BL/6J homozygous embryos. Embryos were transferred to pseudo-pregnant female recipients 2 days after pronuclear injection at the 4-cell stage. Genomic DNA of founder mice was isolated from toe clips, PCR-amplified, cloned into the pCR4Blunt-TOPO vector (Invitrogen, 450031), and Sanger-sequenced to identify mutations. Founder mice were backcrossed to C57BL/6J mice for ≥4 generations and heterozygous mice were bred to homozygosity. Genotyping was performed by PCR on genomic DNA isolated from mouse tails. Primers used for cloning and genotyping are listed in **Table S2**.

Total RNA extraction, RT-PCR, and 5' RACE

Tissues were dissected from adult male mice, washed in phosphate-buffered saline (PBS), frozen in liquid nitrogen and stored at -80°C prior to RNA extraction. Total RNA was extracted using TRIzol according to manufacturer's instructions (Invitrogen). 1 µg of total RNA was reverse-transcribed using the SuperScript IV First-Strand Synthesis System (Invitrogen, 18091050) and synthesized cDNA was PCR-amplified. For 5' RACE, 4 µg of total RNA from testes was reverse-transcribed using 5' RACE System for Rapid Amplification of cDNA Ends (Invitrogen, 18374058) and synthesized cDNA was PCR-amplified, cloned into the pCR4-TOPO TA vector (Invitrogen, K457501) and Sanger-sequenced to identify translation initiation site. Primers used are listed in **Table S2**.

Yeast two-hybrid assays

Full-length mouse *Rnf212b* and *Rnf212* cDNAs were PCR amplified from mouse testis cDNA, prepared as above, and cloned into pGADT7 and pGBKT7 vectors (Clontech) using a Gibson Assembly Cloning Kit (NEB, E2611S). Full-length mouse *Hei10* coding sequence was synthesized by Twist Bioscience and cloned into pGADT7 and pGBKT7. Prey and bait vectors were transformed into the Y187 and Y2HGold strains (Clontech). Transformants were mated on SD/-Trp/-Leu plates. After incubation of the plates at 30°C for 3 days, single colonies were inoculated into SD/-Trp/-Leu media and grown overnight. Colonies were resuspended at OD_{600} = 1, four 10-fold serial dilutions were prepared, and 10 µl drops were spotted onto selection plates and incubated at 30°C for 3 to 5 days. Selection plates are: SD/-Trp/-Leu containing 200 ng/ml of Aureobasidin A (Clontech, 630466) to select for the *AUR1-C* reporter gene, or SD/-Trp/-Leu/-Ade/-His to select for *ADE2* and *HIS3* reporters.

Primers used for cloning and point mutagenesis are listed in **Table S2**.

Antibody production

Polyclonal antibodies against mouse RNF212B were raised in Guinea pigs. Codon-optimized of full-length mouse RNF212B was cloned into pET-28b (+) (Addgene) with a C-terminal 6xHis tag. *E. coli* Arctic Express (DE3) cells (Agilent Technologies Inc.) transformed with the mouse RNF212B-6xHis expression vector were grown in 2L of LB at 30°C to an OD_{600} of 0.8, and protein expression was induced with 0.5 mM IPTG followed by incubation at 11°C in media supplemented with 0.1 mM $ZnCl_2$. ~5 g of cells were pelleted by centrifugation, suspended in 60

ml of denaturing lysis buffer A (6M Guanidine, 25 mM sodium-phosphate pH 7.4, 500 mM NaCl, 0.1 mM $ZnCl_2$, 1 mM β-mercaptoethanol, 20 mM imidazole, 10% glycerol) and sonicated. Samples were centrifuged at 35,000 rpm in a Ti-45 rotor (Beckman) for 45 min and soluble extract was applied to a 5 ml HisTrap FF column (GE healthcare Inc.) using a GE AKTA Avant 25 FPLC system. After equilibrating with 5 column volumes (CV) of lysis buffer A, followed by washes with 10 CV of wash buffer B (6M urea, 25 mM sodium-phosphate pH 7.4, 500 mM NaCl, 1 mM β-mercaptoethanol, 10% glycerol) supplemented with 60 mM imidazole, bound proteins were eluted with a linear gradient of imidazole (60-600 mM) in buffer B. Peak fractions were pooled and dialyzed extensively in decreasing urea concentrations (6M to 1M urea in 25 mM sodium-phosphate pH 7.4, 500 mM NaCl, 1 mM β-mercaptoethanol, 10% glycerol). Protein concentrations were determined by Bradford and spectrophotometric (A_{280}) methods. Two Guinea pigs were immunized with purified RNF212B protein by Antibodies Incorporated (Davis, CA). The IgG fraction was purified from the resultant sera, using the Montage antibody purification kit according to manufacturer's instructions (Millipore Sigma), and dialyzed against PBS containing 10% glycerol and aliquots stored at -80°C.

Histology

Testes from adult male mice were dissected, punctured with a 27-guage needle, and fixed in 10% buffered formalin overnight at room temperature. Ovaries from 18 days postpartum (18 dpp) female mice were dissected and fixed in 10% buffered formalin overnight at room temperature. Fixed testes and ovaries were washed in PBS twice and stored in 70% ethanol at 4°C prior to embedding. Tissues embedded in paraffin were sectioned (5 µm) onto glass slides (Fisher Scientific, 12-550-15), deparaffinized, rehydrated, and incubated with antigen retrieval buffer (10 mM Sodium Citrate, 0.05% Tween-20) for 50 min at 100°C. Slides were then stained with hematoxylin and eosin (testes), or immunostained with anti-p63 antibody, as described below, and counterstained with hematoxylin (ovaries), and mounted with Permount (Fisher Scientific, SP15-100).

Surface spreads of spermatocyte chromosomes

Surface-spread chromosomes of spermatocytes were prepared as described previously (7-9) with slight modification. Testes were dissected, the tunica albuginea removed, and adherent extratubular tissues removed by rinsing and dissociating seminiferous tubules in PBS using a pair of 25-gauge (adult) or 27-guage (juvenile) needles in a 35 mm petri dish (Corning, 430165). Dissociated seminiferous tubules were incubated in hypotonic extraction buffer (30 mM Tris-HCl pH 8.0, 50 mM sucrose, 17 mM trisodium citrate dihydrate, 5 mM EDTA, 0.5 mM dithiothreitol (DTT) and 0.5 mM phenylmethylsulphonyl fluoride (PMSF), pH 8.2-8.3) for 15-45 min at room temperature. After quickly rinsing seminiferous tubules in 100 mM sucrose, a small amount of tubules were placed in 40 µl of 100 mM sucrose on a glass depression slide. Tubules were torn to pieces using two fine forceps, and large pieces of tubular remnant were removed. The volume was increased to 40-60 µl by adding 100 mM sucrose and a cell suspension was made by pipetting. A clean glass slide (Fisher Scientific, 12-544-7) was dipped into freshly made PFA solution (1% paraformaldehyde adjusted to pH 9.2 using 1.25 M sodium borate, 0.15% Triton X-100) in a 50 ml Falcon tube, and excess solution was drained onto a paper towel. 20 µl of cell suspension was placed at the upper right corner of the slide and slowly dispersed in horizontal and vertical directions to homogeneously cover the slide. Hot tap water was added to a humid chamber and slides were slowly dried in the closed chamber overnight at room temperature. Slides were further dried for 3 hr with lid ajar, and then for 1-2 hr with the lid removed. Slides were washed once for 5 min in deionized water and twice for 5 min in 0.4% Photo-Flo 200 solution (Kodak, 1464510) in a coplin jar before air-drying at room temperature. Slides were either directly processed for immunostaining, as described below, or stored wrapped in aluminum foil at -80°C prior to immunostaining. For SIM imaging, 15 µl of cell suspension was placed at the upper right corner of a coverslip (Fisher Scientific, 12-544-E) coated with 90 µl of 1% PFA, 0.15% Triton X-100 solution and slowly dispersed as above.

Surface spreads of oocyte chromosomes

Surface-spread oocyte chromosomes were prepared as described for spermatocytes with modifications. Ovaries from fetal females were dissected into PBS and kept on ice prior to incubation in hypotonic extraction buffer. A pair of ovaries were incubated in hypotonic extraction buffer for 10 min at room temperature, quickly rinsed in 100 mM sucrose, and placed in 50 µl of 100 mM sucrose on a glass depression slide. Ovaries were torn to pieces using a pair of 25-guage needles, and large pieces of ovarian remnant were removed. The volume was increased to 40 µl by adding 100 mM sucrose and a cell suspension was made by pipetting. One half of a clean glass slide was coated with 50 µl of freshly made 1% PFA 0.15% Triton X-100, and 10 µl of cells suspension was placed at the upper right corner of the slide slowly dispersed in horizontal and vertical directions to homogeneously cover the half slide. Slides were then dried, washed, and stored as described for spermatocyte chromosomes spreads.

Chromosome spreads of diakinesis/metaphase-I spermatocytes
Chromosome spreads of diakinesis/metaphase-I spermatocytes were prepared as described previously (9). Testes from adult male mice were dissected, tunica albuginea removed, and seminiferous tubules placed in 2 ml of hypotonic solution (1% trisodium citrate) in a 35 mm petri dish. Seminiferous tubules were torn to pieces using two fine forceps and, after adding 1 ml of hypotonic solution, 3 ml of tubule suspension was transferred to a 15 ml Falcon tube using a plastic transfer pipette (Phenix, PP-137030). Tubule fragments were allowed to settle out for 3 min and the supernatant containing suspended cells was transferred to another 15 ml Falcon tube. Remnant tubule fragments in the petri dish were suspended in 2 ml of hypotonic solution and transferred to the first 15 ml Falcon tube containing tubule fragments. The mixture of tubule fragments was resuspended using a transfer pipette, tubule fragments were settled out, and the supernatant was transferred to the 15 ml Falcon tube containing the first supernatant. The mixture of supernatants was filtered through 70 µm and 40 µm Cell Strainers (Corning 352350; Celltreat, 229481) and suspended cells were pelleted by centrifugation at 900 rpm for 10 min at room temperature. Cells were fixed by adding 3 ml of freshly made fixative solution 1 (75% methanol, 25% acetic acid with 0.375% chloroform) drop-by-drop while gentle vortexing. Cells pelleted by centrifugation at 900 rpm for 10 min at room temperature, resuspended in 3 ml of freshly made fixative solution 2 (75% methanol, 25% acetic acid, chilled to -20°C), pelleted again, and resuspended in 0.5 ml of fixative solution 2. Fixed cell suspension was dropped onto a clean glass slide (Fisher Scientific, 12-544-7) from a height of 2-3 feet height using a glass Pasteur pipette in a room with >40% humidity. Slides were air-dried for 10 min at room temperature and either stained immediately with ProLong Gold or Diamond Antifade Mountant (Thermo Fisher Scientific, P36930 or P36970) containing 1 µg/ml DAPI, or stored at 4°C prior to staining.

Chromosome spreads of metaphase-I oocytes
Chromosome spreads of metaphase-I oocytes were prepared as described previously (9). Ovaries from adult female mice without prior hormonal stimulation were dissected, follicles were punctured in pre-warmed (37°C) M2 media (Sigma-Aldrich, M7167) using a 25-gauge needle, and germinal-vesicle stage oocytes with integral cumulus cell layers were collected. Surrounding cumulus cells were mechanically removed by pipetting and oocytes were cultured in M2 media for 7 hr at 37°C. Metaphase-I oocytes were transferred into Tyrode's acidic solution (Sigma-Aldrich, T1788) to remove the zona pellucida (ZP), and ZP-free oocytes were transferred and maintained in M2 media prior to spreading. 5-10 µl of 1% PFA solution pH 9.2 containing 0.15% Triton X-100 and 3 mM DTT was placed in each well of a 12-well glass slide (Electron Microscopy Sciences, 63425-05) and one ZP-free oocyte was transferred to each well. Slides were air-dried overnight at room temperature, and either directly processed for immunostaining or stored at 4°C for up to several days prior to immunostaining.

Immunostaining
Slides were rehydrated with Tris-buffered saline (TBS) pH 8.0 containing 0.05% of Triton X-100 (TBST) for 3 min, blocked twice with blocking buffer (1% normal goat or donkey serum, 3% bovine serum albumin (BSA), 1x TBS pH 8.0, 0.05% Triton X-100, 0.05% sodium azide) for 15 min at room temperature and incubated with primary antibodies in antibody dilution buffer (10% normal goat or donkey serum, 3% BSA, 1x TBS pH 8.0, 0.05% Triton X-100, 0.05% sodium

azide) in a humid chamber overnight at room temperature. Slides were briefly rinsed with TBST, washed twice with TBST for 5 min, blocked twice with blocking buffer for 15 min at room temperature and then incubated with secondary antibodies in antibody dilution buffer in a humid chamber for 1 hr at 37°C. Slides were rinsed with TBST, washed three times with TBST for 5 min, washed once with Milli-Q water for 2 min, and air-dried prior to mounting with ProLong Gold or Diamond Antifade Mountant. For SIM imaging, cover slips were blocked three times for 15 min prior to primary antibody incubation.

For metaphase-I oocytes, slides were briefly rehydrated with TBST, washed three times with TBST for 5 min, blocked twice with blocking buffer for 15 min at room temperature, and incubated with primary antibodies in antibody dilution buffer in a humid chamber overnight at room temperature. Slides were rinsed with TBST, briefly washed twice with TBST, blocked twice with blocking buffer for 15 min at room temperature, and incubated with secondary antibodies in antibody dilution buffer in a humid chamber for 1 hr at room temperature. Slides were rinsed with TBST, briefly washed with TBST, and then washed three times with TBST for 5 min. Slides were then incubated with TBST containing 5 μg/ml DAPI for 10 min at room temperature and mounted with 50% ProLong Gold or Diamond Antifade Mountant in TBST.
All primary and secondary antibodies used are listed in **Table S3**.

Protein blot analysis
Testes were dissected from 16-18 dpp juvenile male mice and frozen in liquid nitrogen. Five pairs of testes were homogenized in 1 mL ice-cold RIPA buffer (50 mM Tris-HCl pH 7.5, 150 mM NaCl, 1 mM EDTA, 1% NP-40, 0.5% sodium deoxycholate, 0.1% sodium dodecyl sulfate, SDS) supplemented with protease and isopeptidase inhibitors (1x Complete protease inhibitor EDTA-free, Roche, 04693159001), 1 mM PMSF, 10 mM N-ethylmaleimide, NEM) , incubated for 15 min on ice, sonicated (20% duty cycle, output 2 in burts for 2.5 min), and then incubated for 15 min on ice. Following centrifugation at 14,000 rpm for 15 min at 4°C, supernatants were collected as whole-testis extracts. Protein concentrations determined by Bradford assay were normalized, and samples were subjected to electrophoresis and immunoblotting. Membranes were blocked with TBST plus 2.5% non-fat milk for 1 hr at room temperature and incubated with primary antibodies overnight at 4°C. Membranes were washed three times with TBST for 10 min each, then incubated with HRP-conjugated secondary antibodies in TBST for 1 hr at room temperature. After four washes with TBST for 10 min each, HRP signal was developed using the SuperSignal West Pico Chemiluminescent Substrate (Thermo Scientific, PI-34080) and detected using an Amersham Imager 600 (GE Healthcare).

All primary and HRP-conjugated secondary antibodies used are listed in **Table S3**.

Image acquisition
Images of surface-spread prophase chromosomes and metaphase-I chromosome spreads were acquired using a Zeiss AxioPlan II microscope with a 63 x Plan-Apochromat 1.4 NA objective and EXFO X-Cite metal halide light source, captured with a Hamamatsu ORCA-ER CCD camera and processed using Volocity (Perkin Elmer) and Photoshop (Adobe) software. SIM images were acquired using a Nikon N-SIM super-resolution microscope and processed using NIS-Elements 2 image processing software. Airyscan images were acquired using a Zeiss LSM800 with Airyscan microscope with a 60 x 1.4 NA objective and processed using ZEN imaging software (Carl Zeiss). Images of testis sections were acquired using a Zeiss Axio Imager M2 microscope with a 20 x Plan-Apochromat 0.8 NA objective, captured with a Hamamatsu ORCA-Flash 4.0 V3 sCMOS camera and processed using ZEN imaging software (Carl Zeiss). Images of ovary sections were acquired using a ScanScope digital scanner (Asperio) and processed using ImageScope software.

Image analysis
Comparisons were made between animals that were either littermates or matched by age. Numbers and colocalization of foci were determined manually. *Rnf212b*$^{-/-}$, *Rnf212*$^{-/-}$ and *Rnf212b*$^{RING/RING}$ mutant analysis was blinded with respect to the genotype of the animals. Results from ≥2 independent experiments/animals were pooled for quantification of number of foci and chiasmata. *Spo11*$^{-/-}$, *Sycp1*$^{-/-}$, *Mlh3*$^{-/-}$ and *Hei10*$^{mei4/mei4}$ mutant analysis was not blinded to

genotype because phenotypes are overt. For focus intensity analyses, RNF212B and RNF212 foci along homolog axes were automatically defined by thresholding intensity on SYCP3 staining and manually inspecting images to separate any adjacent focus pairs defined as one focus. For *Spo11*$^{-/-}$ mutant analysis, synapsed regions marked by SYCP1 were manually cropped and intensities of RNF212B and RNF212 were measured. Two regions of interest (ROI) were drawn inside nuclei but adjacent to homolog axes and their average intensity was subtracted as background for each nucleus. Results from one experiment/animal among ≥2 independent experiments/animals were shown for quantification of intensities.

Prophase-I stages were defined by SYCP3 staining (spermatocytes) and both SYCP3 staining and fetal age (oocytes) using standard criteria. Leptonema was defined by short SYCP3 stretches without evidence of synapsis determined by thickening of SYCP3 staining. Zygonema was defined by longer stretches of SYCP3 with various degree of synapsis: early, mid- and late-zygonema were defined by having <25%, 25-75% and >75% of synapsis, respectively. Pachynema was defined by full synapsis of all autosomes. For spermatocytes, early pachynema was defined by extensive synapsis between the X-Y chromosomes, mid pachynema was defined by limited/end-to-end X-Y synapsis, and late pachynema was defined by decondensed/elongated X-Y chromosomes with figure-of eight configuration and thickening of SYCP3 staining at telomeres of autosomes. Diplonema in spermatocytes was defined by internal desynapsis with various degrees of residual synapsis: early, mid and late diplonema were defined by having >75%, 75-25% and<25% of synapsis, respectively. For oocytes, nuclei with unsynapsed/desynapsed chromosomes at embryonic day 15.5 (E15.5) and at 0.5 day postpartum (0.5 dpp) were defined as zygonema and diplonema, respectively. Dictyate stage was defined by short, ragged SYCP3 stretches without evidence of synapsis at 0.5 dpp.

For ovary sections, numbers of oocytes were manually counted for every fifth section and counts were multiplied by five to calculate the total number of oocytes per pair of ovaries, as described previously (10).

Supplemental Figures

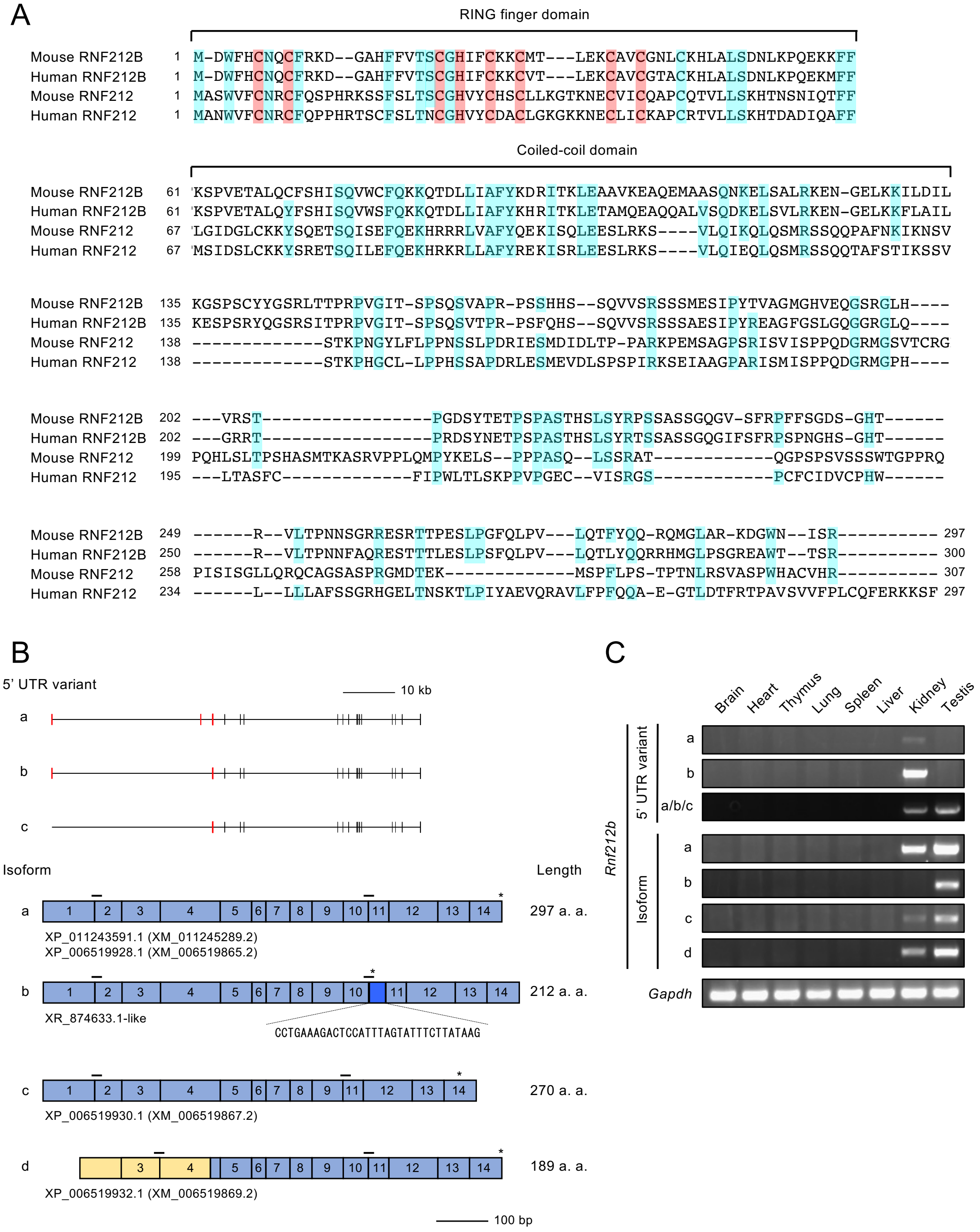

Fig. S1. 5' UTR variants and isoforms of mouse *Rnf212b*.

(A) Protein sequence alignments of mouse and human RNF212B and RNF212 full-length isoforms. Zinc-coordinating cysteine and histidine (pink) residues of RING finger domain and other identical residues (blue) are highlighted.

(B) Schematic of 5' UTR variants and isoforms of mouse *Rnf212b*. Top, red and black bars represent 5' untranslated exons (5' UTRs) and coding exons, respectively. Two 5' UTR variants

were identified in the NCBI database, *variants a* and *b*, which differ by a deletion of the second untranslated exon in *variant b*. A third *variant c* was identified by 5' RACE from testes and comprises ~1/3 of the third untranslated exon of *variant a* followed by the 14 coding exons. Bottom, yellow and blue boxes represent 5' UTRs and coding exons, respectively. Four different coding isoforms were identified in the NCBI database. For simplicity, 5' UTRs of isoforms a-c are not shown. Isoform a encodes the full-length RNF212B protein of 297 amino acids; isoform b uses an alternative splice acceptor site, creating a premature stop codon after exon 10 that is predicted to produce a protein lacking 86 C-terminal amino acids; isoform c uses an alternative splice donor site to precisely skip exon 10, which corresponds to 16 amino acids of the disordered serine-rich C-terminal tail, resulting in a predicted protein of 270 amino acids; and isoform d lacks the first two exons present in other isoforms and uses an extended exon 3 and most of exon 4 as a 5' UTR, to encode a predicted protein of 189 amino acids that lacks the N-terminal RING finger domain. Asterisks indicate positions of stop codons. Horizontal bars represent positions of primers used to detect each isoform by RT-PCR in (C). Annotated or related variants and isoforms in the NCBI database are also shown. Expression of full-length a and b isoforms in testis was confirmed by *de novo* cloning from testis mRNA (the sequence of cloned isoform b was similar but not identical to XR_874633).
(C) Expression of mouse *Rnf212b* 5' UTR variants and isoforms analyzed by RT-PCR. Total RNA was extracted from indicated tissues of mature male mice and subjected to RT-PCR. *Gapdh* is a loading control.

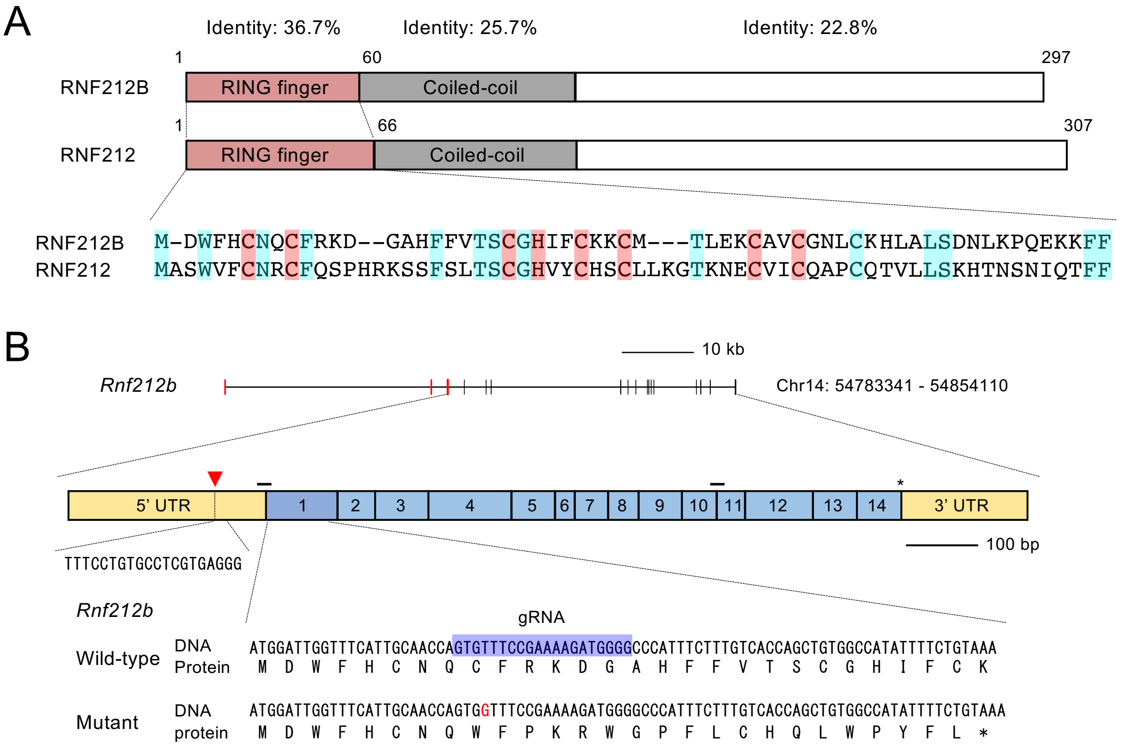

Fig. S2. Domain structure of RNF212B and strategy to generate *Rnf212b* mutant mice by CRISPR/Cas9.
(A) Domain structures of mouse RNF212B and RNF212. Amino acid sequences of the RING finger domains are shown below. Presumptive zinc-coordinating cysteines and histidines and other identical residues are highlighted in pink and blue, respectively. The domain structure of the full-length RNF212B protein mirrors that of RNF212 with an N-terminal RING finger domain (36.7% amino-acid identity with RNF212), a ~70 amino acid region of predicted coiled-coil (25.7% identical) and a disordered serine-rich C-terminal tail (22.8% identical).
(B) Schematic of the mouse *Rnf212b* gene on chromosome 14. Top row: red and black bars represent 5’ untranslated exons (5’ UTRs) and coding exons, respectively. Middle row: yellow and blue boxes represent 5’ UTRs and coding exons, respectively, in the full-length isoform a. The inverted red triangle represents the translation initiation site in testis identified by 5’ RACE. An asterisk represents the stop codon. Horizontal bars represent the positions of primers used to detect 5’ UTR variants a/b/c by RT-PCR in **Figure S1C**. Bottom row: nucleotide and amino-acid sequences at the CRISPR/Cas9 targeted site in exon 1. A single-nucleotide insertion (shown in red) created a frameshift and premature stop codon (asterisk). The guide RNA (gRNA) sequence is highlighted in blue.

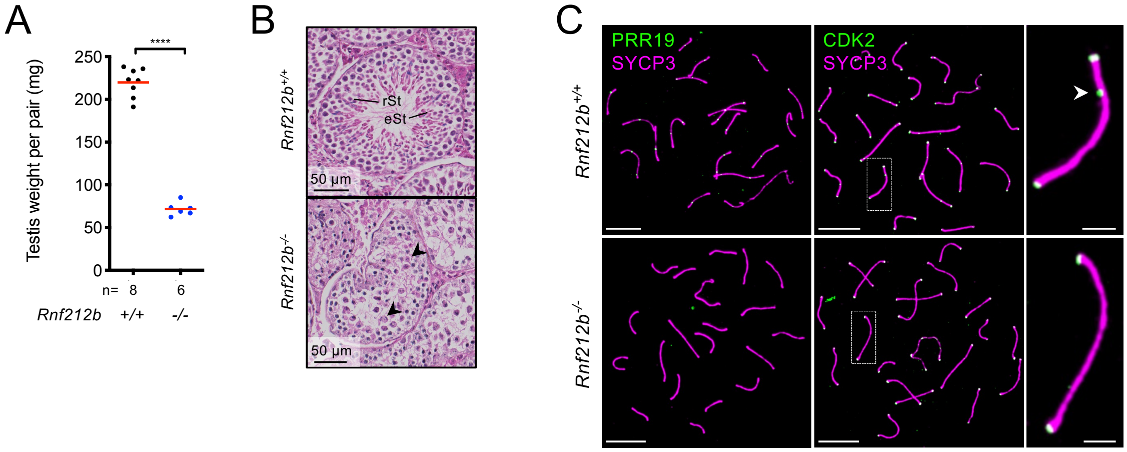

Fig. S3. Infertility of *Rnf212b*$^{-/-}$ mutant male mice due to crossover failure.
(A) Smaller testes in *Rnf212b*$^{-/-}$ males. Red bars indicate means. ****$p \leq 0.0001$, two-tailed *t* test. n, numbers of mice analyzed.
(B) Defective spermatogenesis in *Rnf212b*$^{-/-}$ males. Seminiferous tubule sections stained with hematoxylin and eosin. rSt, round spermatid; eSt, elongated spermatid. Arrowheads indicate metaphase cells.
(C) Crossover-specific immunostaining foci of PRR19 (left) and CDK2 (right) foci were absent from chromosome spreads of *Rnf212b*$^{-/-}$ spermatocytes. Mid/late pachytene nuclei from wild-type and *Rnf212b*$^{-/-}$ spermatocytes were immunostained for SYCP3 plus PRR19 (left) or CDK2 (right). The magnified images show representative chromosomes from the CDK2 staining. Note that CDK2 also localizes to telomeres, which is not affected by *Rnf212b* mutation. An arrowhead indicates an interstitial CDK2 focus marking a prospective crossover site. Scale bars, 10 µm for images of full nuclei and 2 µm for magnified panels.

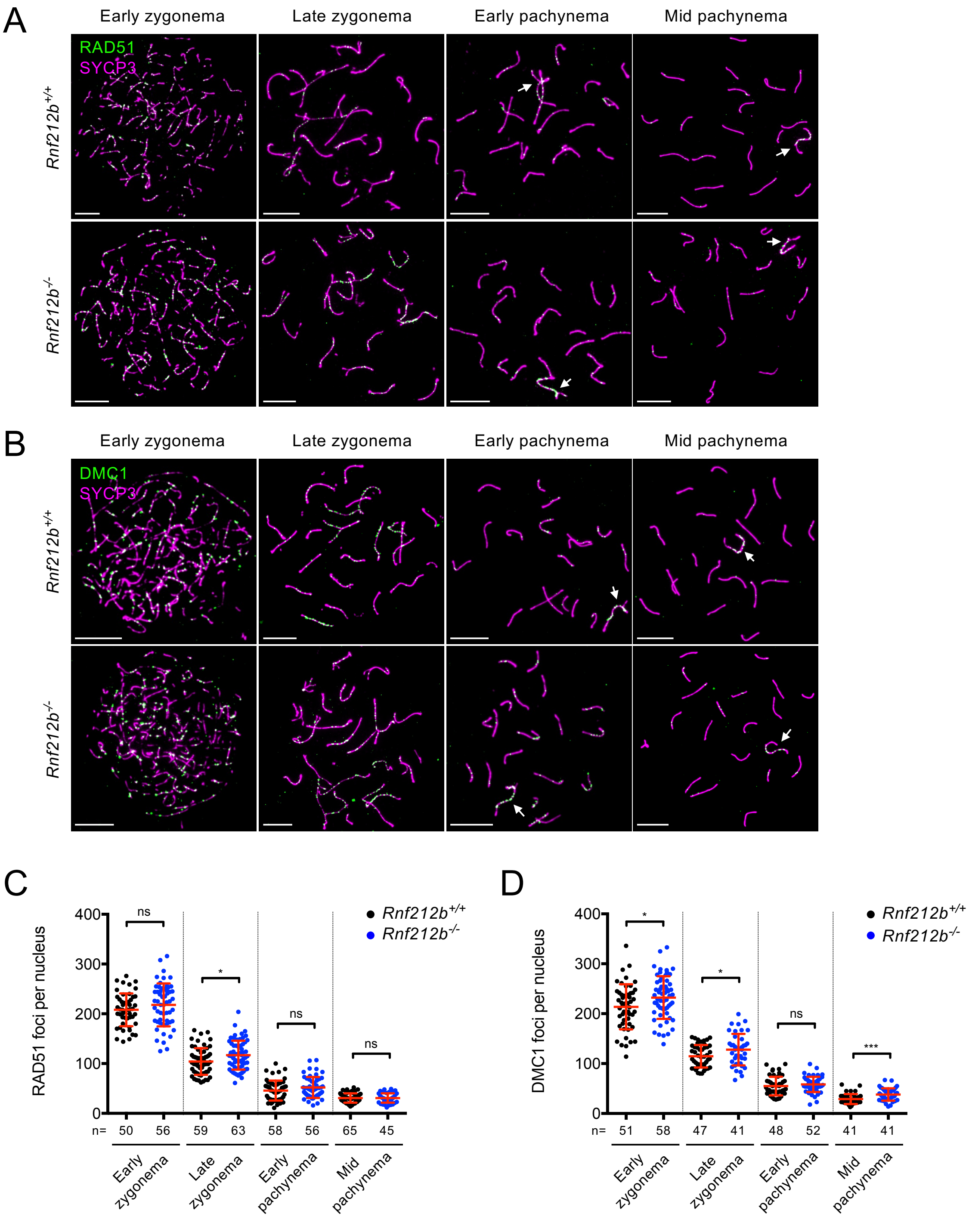

Fig. S4. Dynamics of RAD51 and DMC1 throughout zygonema and pachynema.
(A and B) Representative images of RAD51 (A) and DMC1 (B) immunostainings in wild-type and *Rnf212b*$^{-/-}$ spermatocytes nuclei immunostained for SYCP3 plus RAD51 (A) or DMC1 (B) at the indicated stages. Arrows indicate the X-Y chromosomes. Scale bars, 10 μm.
(C and D) Focus counts of RAD51 (C) and DMC1 (D). Red bars indicate means ± SDs.
ns, not significant (p > 0.05); *p ≤ 0.05; ***p ≤ 0.001 for two-tailed Mann-Whitney tests. Total numbers of nuclei analyzed are indicated below the X axes.

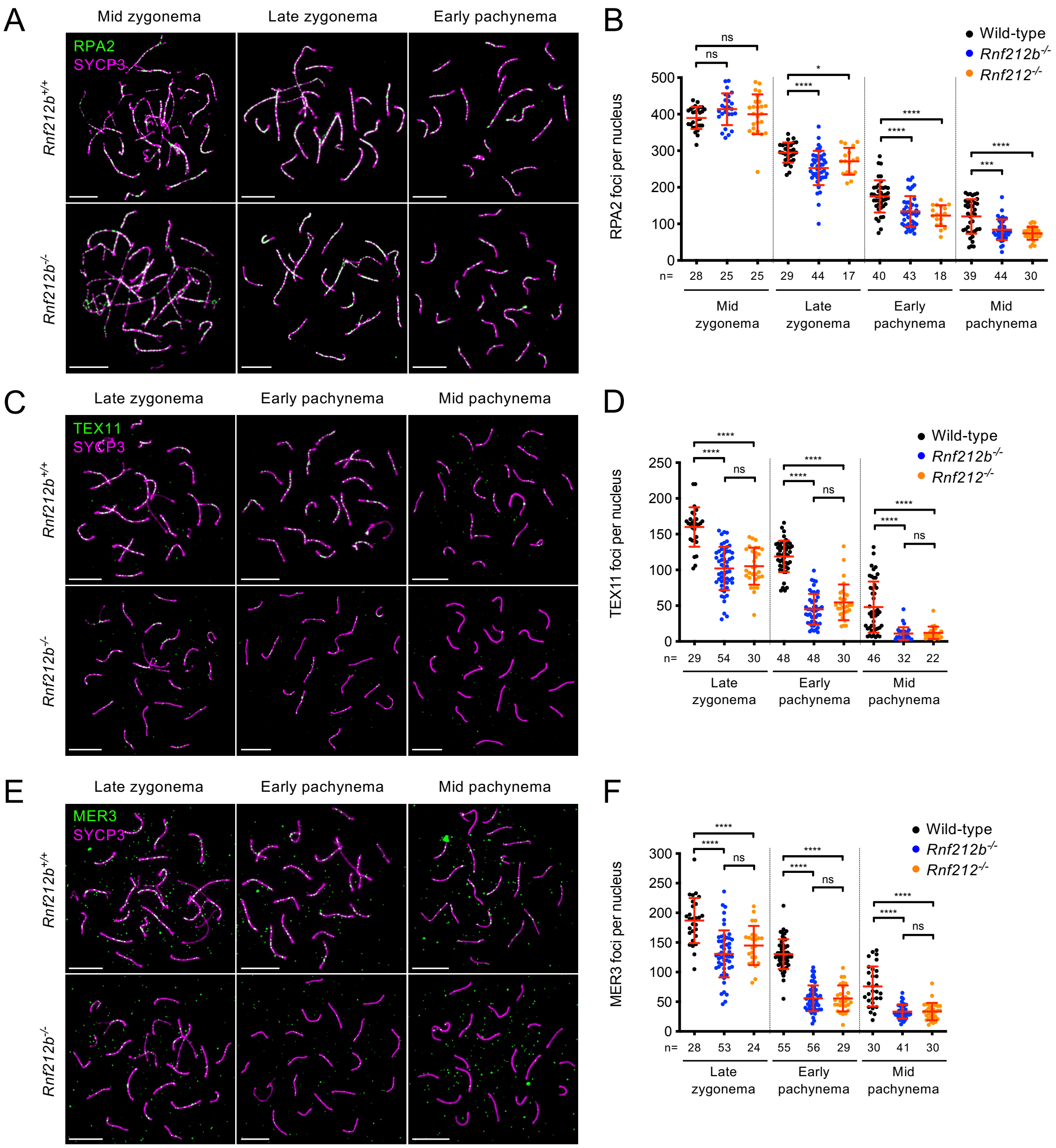

Fig. S5. Intermediate steps of recombination are perturbed in *Rnf212b*$^{-/-}$ mutant mice.
(A, C and E) Images of wild-type and *Rnf212b*$^{-/-}$ spermatocyte nuclei immunostained for SYCP3 plus RPA2 (A), TEX11 (C) or MER3 (E) at the indicated stages. Scale bars, 10 µm.
(B, D and F) Counts of RPA2 (B), TEX11 (D) and MER3 (F) immunostaining foci. Red bars indicate means ± SDs. ns, not significant ($p > 0.05$); *$p \leq 0.05$; ***$p \leq 0.001$; ****$p \leq 0.0001$ for two-tailed Mann-Whitney tests. Total numbers of nuclei analyzed are indicated below the X axes.

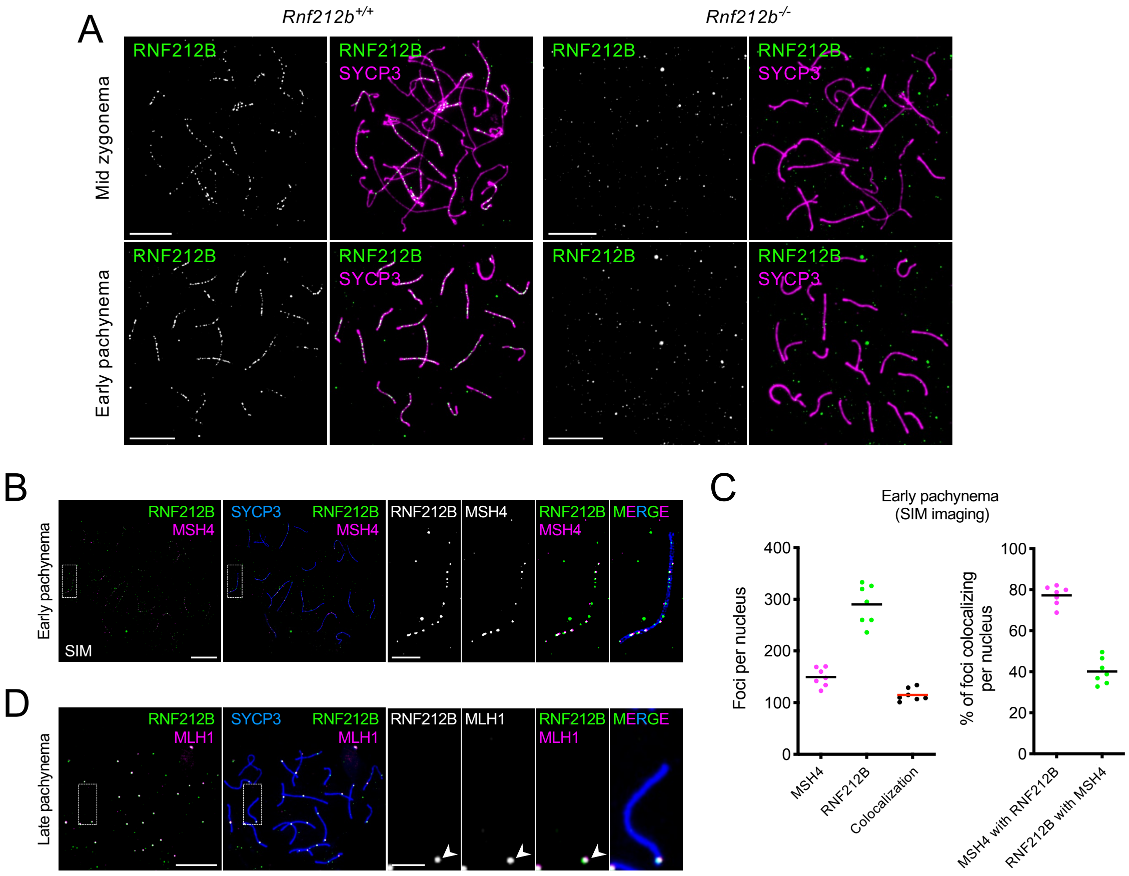

Fig. S6. Chromosomal localization of RNF212B in mouse spermatocytes.

(A) Specificity of anti-RNF212B antibody. Wild-type and *Rnf212b*$^{-/-}$ spermatocyte nuclei at indicated stages were immunostained for SYCP3 and RNF212B. Scale bars, 10 µm.

(B) RNF212B-MSH4 colocalization in SIM image of an early pachytene spermatocyte immunostained for SYCP3, RNF212B and MSH4.

(C) Quantification of RNF212B-MSH4 colocalization. Left, focus counts. Right, degree of colocalization. Black and red bars indicate means. SIM images of 7 early pachytene nuclei were analyzed. RNF212B foci were in 2-fold excess over MSH4 (290.0 ± 38.3 RNF212B versus 149.4 ± 17.8 MSH4 foci per nucleus; means ± SDs; n = 7).

(D) RNF212B-MLH1 colocalization in a late pachytene spermatocyte immunostained for SYCP3, RNF212B and MLH1. Arrowheads indicate crossover sites.

Magnified images in (B) and (D) show representative chromosomes. Scale bars, 10 µm for full nuclei and 2 µm for magnified images.

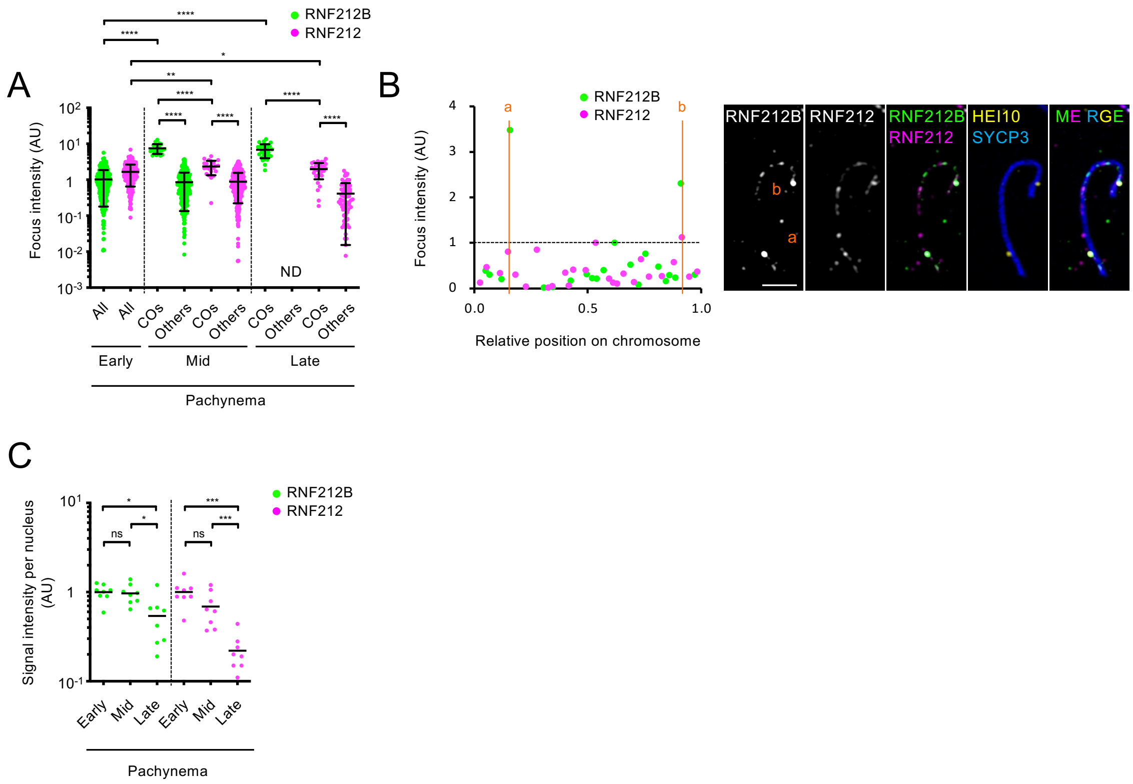

Fig. S7. Distinct localization patterns of differentiating RNF212B and RNF212 crossover foci.

(A) Quantification of focus intensities for RNF212B and RNF212. Each dot represents each focus of RNF212B and RNF212 from the early, mid and late pachytene nuclei (one nucleus per stage) analyzed in **Figure 3D**. Bars indicate means ± SDs. All, all foci; COs, crossover-specific foci that colocalize with HEI10 foci; Others, foci that don't colocalize with HEI10 foci (noncrossover foci). ND, not detected. **p* ≤ 0.05; ***p* ≤ 0.01; *****p* ≤ 0.0001 for two-tailed Mann-Whitney tests.

(B) Representation of per chromosome analysis of RNF212B and RNF212 foci for a mid-pachytene chromosome (shown in the images in the right-hand panels). Each focus of RNF212B or RNF212 is represented by a dot. The dashed line indicates the signal intensity of the brightest other focus (noncrossover focus) along the same chromosome. Orange vertical lines indicate the positions of HEI10-positive crossover sites (a) and (b). Intensities of foci at these sites relative to that of the brightest other (noncrossover) focus are: (a) 3.5 and (b) 2.3 for RNF212B; (a) 0.8 and (b) 1.1 for RNF212.

(C) Total signal intensity of RNF212B and RNF212 per nucleus in early, mid and late pachytene spermatocytes. Black bars indicate means. ns, not significant ($p > 0.05$); **p* ≤ 0.05; ****p* ≤ 0.001 for two-tailed Mann-Whitney tests. Nuclei analyzed were the same as in **Figure 3D**.

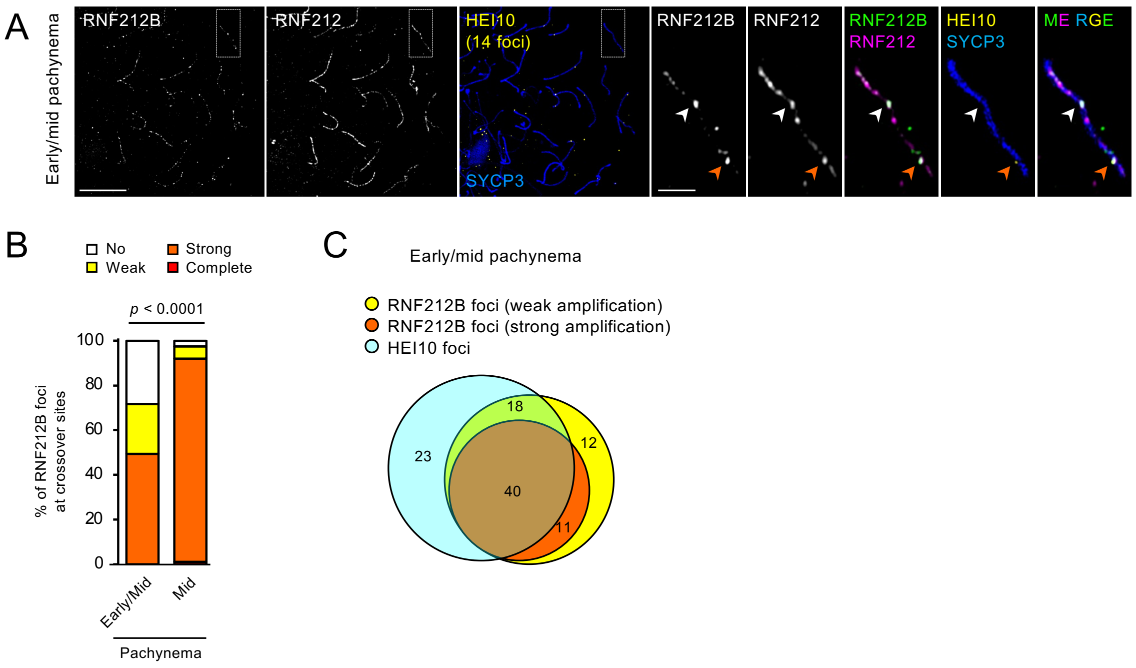

Fig. S8. Timing of RNF212B focus differentiation and appearance of HEI10 foci in spermatocytes.

(A) Differentiation of RNF212B foci relative to the emergence of HEI10 foci. Airyscan images of an early-to-mid pachytene spermatocyte nucleus immunostained for SYCP3, RNF212B, RNF212 and HEI10. This nucleus contains 14 HEI10 foci indicating that it is in transition to full differentiation of crossovers sites (≥20 HEI10 foci). The white arrowhead indicates a differentiated RNF212B focus without an associated HEI10 focus; and the orange arrowhead indicates a differentiated RNF212B focus associated without a small, emerging HEI10 focus. These configurations indicate that growth of RNF212B foci can precede the emergence of HEI10 foci.

(B) Differentiation of RNF212B at crossover sites. Crossover-specific foci of RNF212B (i.e. RNF212B foci that colocalized with HEI10 foci) in early-to-mid pachytene nuclei (containing 5-18 HEI10 foci), and mid-pachytene nuclei (≥19 HEI10 foci) were classified based on their degree of differentiation by measuring their intensity relative to the brightest other (noncrossover) RNF212B focus along the same chromosome (complete differentiation means no other foci detected along the SC; strong differentiation, ≥2-fold brighter; weak differentiation, 1.5-2-fold brighter; or no differentiation, <1.5-fold brighter). 81 crossover-specific RNF212B foci from 7 early-to-mid pachytene and 186 crossover-specific RNF212B foci from 8 mid-pachytene nuclei were analyzed by Airyscan imaging. In early-mid pachytene nuclei, the majority of HEI10 foci are associated with detectable differentiation of RNF212B but 28% are not.

(C) Differentiation of RNF212B and emergence of HEI10 foci in early-to-mid pachynema. Numbers of HEI10 foci (sky blue), RNF212B foci with weak differentiation (yellow) and RNF212B foci with strong differentiation (orange) are shown. Criteria for RNF212B focus differentiation are as in (B). 71.6% (58/81 foci) of sites marked by HEI10 were coincident with weak or strong differentiation of RNF212B, while 78.4% (40/51 foci) of strongly differentiated RNF212B foci colocalized with a HEI10 focus. Thus, with respect to the differentiation of RNF212B and the emergence of HEI10 at prospective crossover sites, a sequential order of events is hard to discern, i.e. these events may be coincident, consistent with the dependence of RNF212B focus differentiation on HEI10. 7 early-to-mid pachytene nuclei with 5-18 HEI10 foci per nucleus were analyzed by Airyscan imaging.

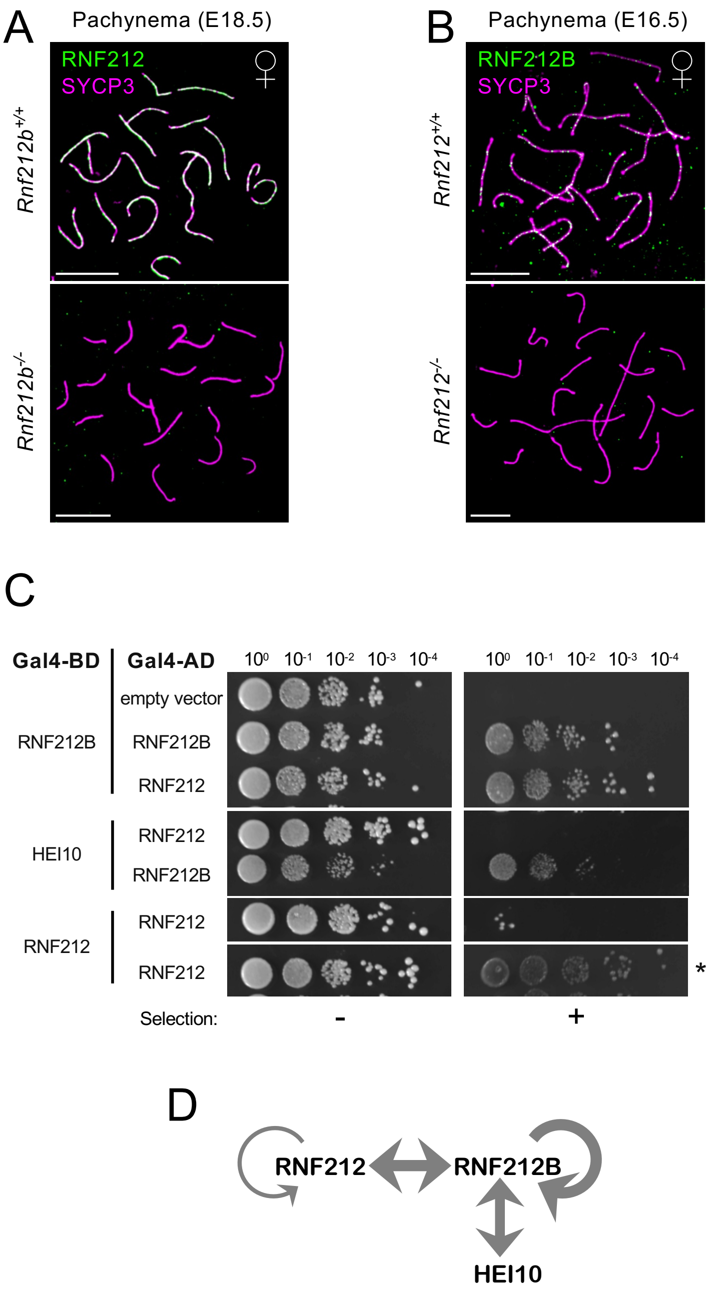

Fig. S9. Relationship between RNF212B, RNF212 and HEI10.
(A and B) RNF212 (A) and RNF212B (B) are interdependent for chromosomal localization in oocytes. Pachytene-stage oocytes at embryonic day 18.5 or 16.5 (E18.5 or E16.5) from the indicated genotypes were immunostained for SYCP3 and RNF212 (A) or RNF212B (B). Scale bars, 10 µm.
(C) Y2H analysis of interactions between RNF212B, RNF212 and HEI10. Yeast cells expressing Gal4 activation (Gal4-AD) and binding (Gal4-BD) domain fused to the indicated proteins, or empty vectors as controls, were spotted at the indicated dilutions. Aureobasidin A selection was used to detect activation of the *AUR1-C* reporter. An asterisk indicates less stringent selection for *ADE2* and *HIS3* reporters.
(D) Summary of Y2H interactions.

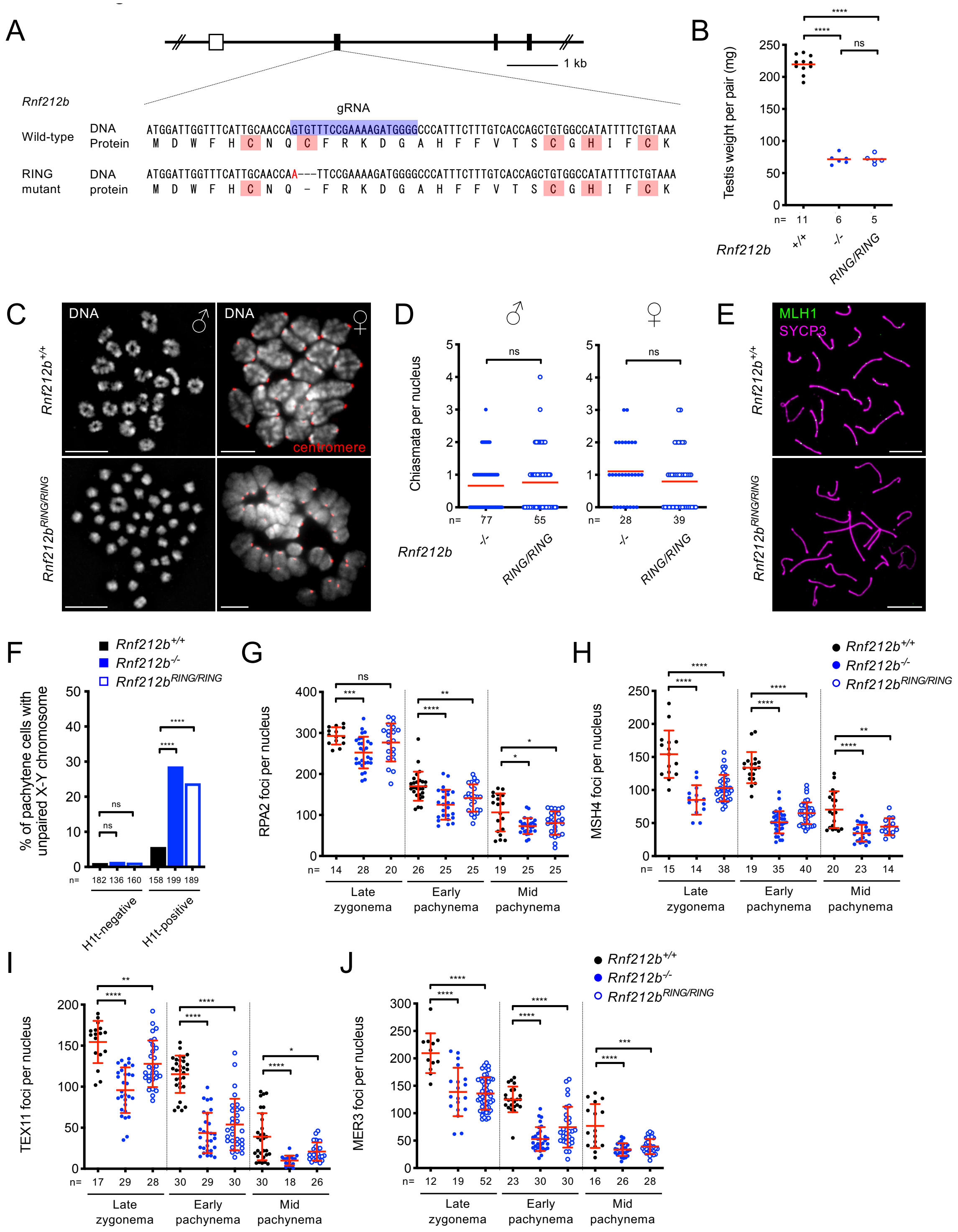

Fig. S10. The RING finger domain is required for RNF212B function *in vivo*.
(A) Scheme of the *Rnf212b* RING mutation. A three-nucleotides deletion in addition to a single-nucleotide substitution (shown in red) induced loss of the 9th cysteine residue with no change in other protein-coding amino acids. White and black boxes indicate 5' UTR and coding exons, respectively. The guide RNA (gRNA) sequence is highlighted in blue. Cysteine and histidine residues of RING finger domain are highlighted in pink.

(B) Reduced testis size in *Rnf212b*$^{RING/RING}$ mutants. Red bars indicate means. ns, not significant (p > 0.05); ****$p \leq 0.0001$ for two-tailed t tests. Numbers of mice analyzed are indicated below the X axis.
(C) Diminished chiasma numbers in *Rnf212b*$^{RING/RING}$ meiocytes. Left, wild-type and *Rnf212b*$^{RING/RING}$ spermatocytes in diakinesis/metaphase-I stages stained with DAPI. Right, metaphase-I oocytes from ≥2 months old wild-type and *Rnf212b*$^{RING/RING}$ females were stained with DAPI and immunostained for centromeres. Scale bars, 10 μm.
(D) Chiasma counts in *Rnf212b*$^{-/-}$ and *Rnf212b*$^{RING/RING}$ meiocytes. Red bars indicate means. ns, not significant (p > 0.05) for two-tailed Mann-Whitney tests. Total numbers of nuclei analyzed are indicated below the X axis.
(E) Absence of MLH1 foci in *Rnf212b*$^{RING/RING}$ spermatocytes. Mid-late pachytene nuclei from wild-type and *Rnf212b*$^{RING/RING}$ spermatocytes immunostained for SYCP3 and MLH1. Scale bars, 10 μm.
(F) High frequency of unpaired X-Y chromosomes in H1t-positive pachytene *Rnf212b*$^{RING/RING}$ cells. ns, not significant (p > 0.05); ****$p \leq 0.0001$ for fisher's exact tests. Total numbers of cells analyzed from 3 mice of each genotype are indicated below the X axis.
(G-J) Focus counts of RPA2 (G), MSH4 (H), TEX11 (I) and MER3 (J) in wild-type, *Rnf212b*$^{-/-}$, and *Rnf212b*$^{RING/RING}$ spermatocytes. Red bars indicate means ± SDs. ns, not significant (p > 0.05); *$p \leq 0.05$; **$p \leq 0.01$; ***$p \leq 0.001$; ****$p \leq 0.0001$ for two-tailed Mann-Whitney tests. Total numbers of nuclei analyzed are indicated below the X axes.

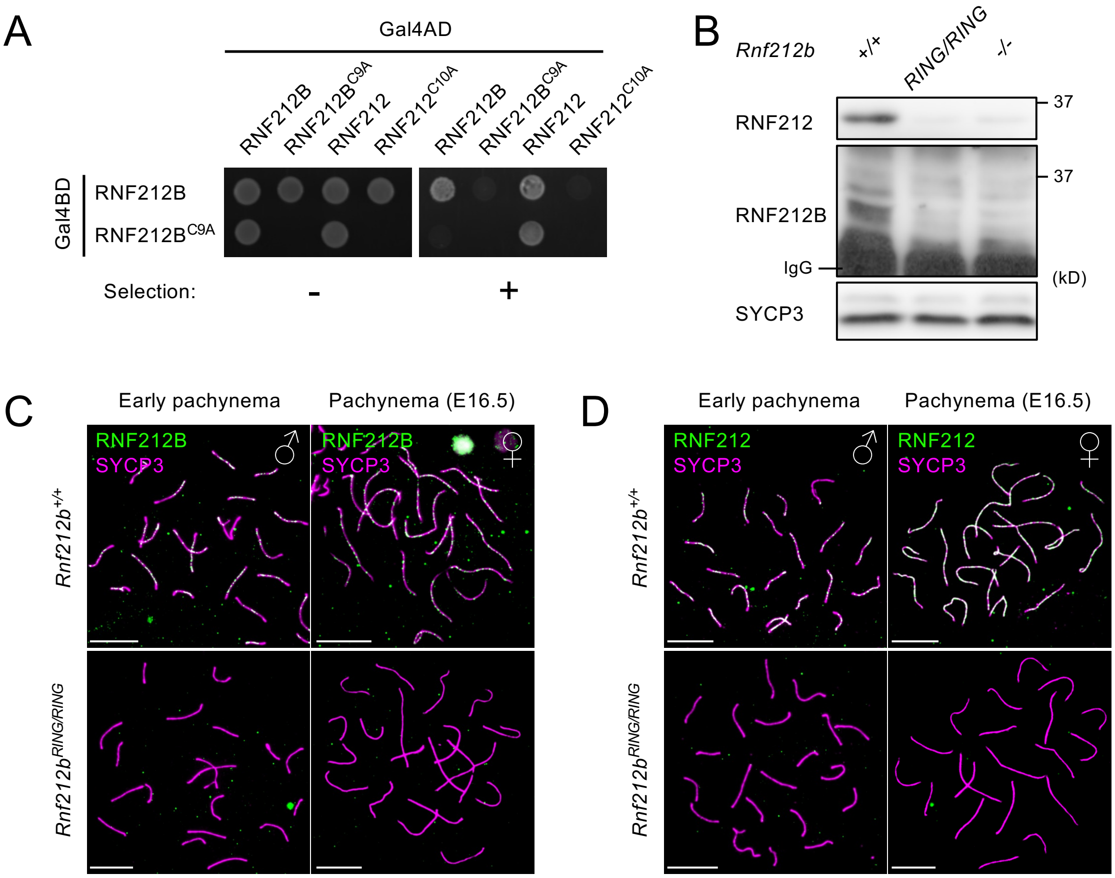

Fig. S11. The role of the RING-finger domain of RNF212B for self-interaction, protein stability and chromosomal localization.

(A) Yeast two-hybrid assay shows that a C9A RING-domain mutation in RNF212B diminishes its self-interaction but not its interaction with RNF212.

(B) Diminished protein levels of RNF212B and RNF212 in *Rnf212b^RING/RING^* spermatocytes. Whole-testis extract from juvenile males (16-18 dpp) of indicated genotypes were subjected to immunoblotting for RNF212 and IP-immunoblotting for RNF212B. SYCP3 is a loading control of meiotic cells.

(C and D) Chromosomal localization of RNF212B (C) and RNF212 (D) is diminished in *Rnf212b^RING/RING^* mutant meiocytes. Early pachytene spermatocyte nuclei from wild-type and *Rnf212b^RING/RING^* testes (left); and pachytene-stage nuclei from wild-type and *Rnf212b^RING/RING^* oocytes at E16.5 (right) were immunostained for SYCP3 and RNF212B (C) or RNF212 (D). Scale bars, 10 μm.

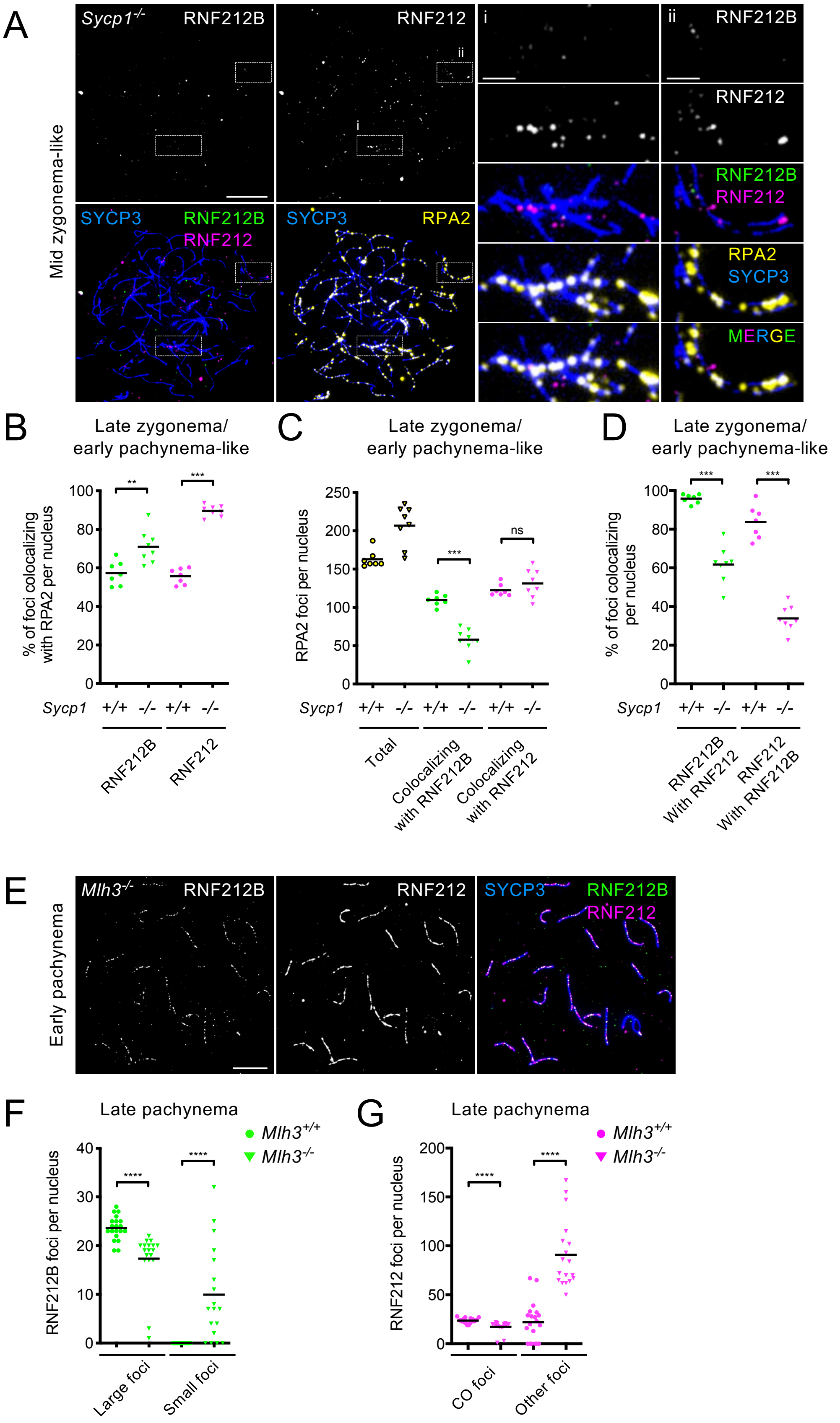
A
Mid zygonema-like
Sycp1-/-
RNF212B
RNF212
i
ii
SYCP3
RNF212B
RNF212
RPA2
MERGE
B
Late zygonema/ early pachynema-like
% of foci colocalizing with RPA2 per nucleus
Sycp1 +/+ -/- +/+ -/-
RNF212B
RNF212
C
Late zygonema/ early pachynema-like
RPA2 foci per nucleus
ns
Total
Colocalizing with RNF212B
Colocalizing with RNF212
D
Late zygonema/ early pachynema-like
% of foci colocalizing per nucleus
RNF212B With RNF212
RNF212 With RNF212B
E
Early pachynema
Mlh3-/-
RNF212B
RNF212
SYCP3
F
Late pachynema
RNF212B foci per nucleus
Mlh3+/+
Mlh3-/-
Large foci
Small foci
G
Late pachynema
RNF212 foci per nucleus
CO foci
Other foci

Fig. S12. Chromosomal localization of RNF212B and RNF212 in mutants defective for synapsis and crossing over.
(A) A mid zygotene-like *Sycp1*$^{-/-}$ spermatocyte nucleus immunostained for SYCP3, RNF212B, RNF212, and RPA2. General staining of RNF212B and RNF212 is absent but limited localization to small regions where homologous axes are closely aligned can be detected (magnified in the panels on the right).
(B and C) Quantification of RPA2 foci colocalizing with RNF212B and RNF212. (B) Degree of RNF212B and RNF212 colocalization with RPA2. (C) Quantification of total and colocalizing RPA2 foci. Black bars indicate means. 7 early-pachytene *Sycp1*$^{+/+}$ nuclei and 8 late-zygotene/early-pachytene-like *Sycp1*$^{-/-}$ nuclei were analyzed.
(D) Quantification of RNF212B-RNF212 colocalization. Black bars indicate means. 7 early pachytene *Sycp1*$^{+/+}$ nuclei and 8 late zygotene/early pachytene-like *Sycp1*$^{-/-}$ nuclei were analyzed.
(E) Normal chromosomal localization of RNF212B and RNF212 in early pachytene *Mlh3*$^{-/-}$ mutant spermatocyte nuclei. An early pachytene nucleus immunostained for SYCP3, RNF212B and RNF212 is shown.
(F) Numbers of RNF212B and RNF212 foci in wild-type and *Mlh3*$^{-/-}$ late-pachytene spermatocyte nuclei. Left, numbers of large and small RNF212B foci. Right, numbers of RNF212 foci associated with crossover sites (colocalizing with large RNF212B foci) and other sites. Black bars indicate means. 20 *Mlh3*$^{+/+}$ and 18 *Mlh3*$^{-/-}$ nuclei were analyzed.
ns, not significant ($p > 0.05$); **$p \leq 0.01$; ***$p \leq 0.001$; ****$p \leq 0.0001$ for two-tailed Mann-Whitney tests. Scale bars, 10 μm in images of full nuclei (A and E) and 2 μm in the magnified panels (A).

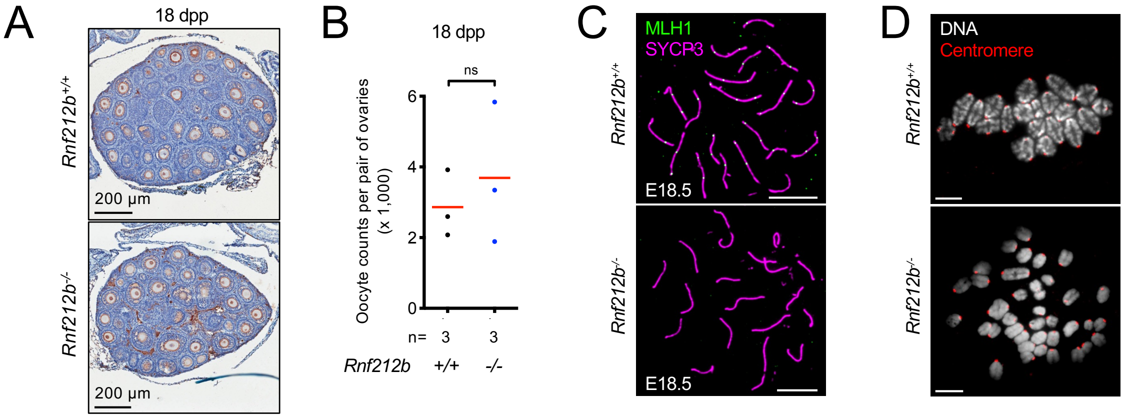

Fig. S13. Infertility of *Rnf212b*$^{-/-}$ mutant female mice due to crossover failure.
(A) Ovary sections from 18 days postpartum (dpp) females immunostained for p63 and counterstained with hematoxylin.
(B) Oocyte counts at 18 dpp. Red bars indicate means. ns, not significant ($p > 0.05$, two-tailed t test). n, numbers of mice analyzed.
(C) MLH1 foci are absent in *Rnf212b*$^{-/-}$ oocytes. Pachytene-stage oocyte nuclei from ovaries of embryonic day 18.5 females immunostained for SYCP3 and MLH1. Scale bars, 10 µm.
(D) Chiasmata are diminished in *Rnf212b*$^{-/-}$ oocytes. Metaphase-I oocytes from ovaries of ≥2-month-old females stained with DAPI and immunostained for CREST (centromeres). Scale bars, 10 µm.

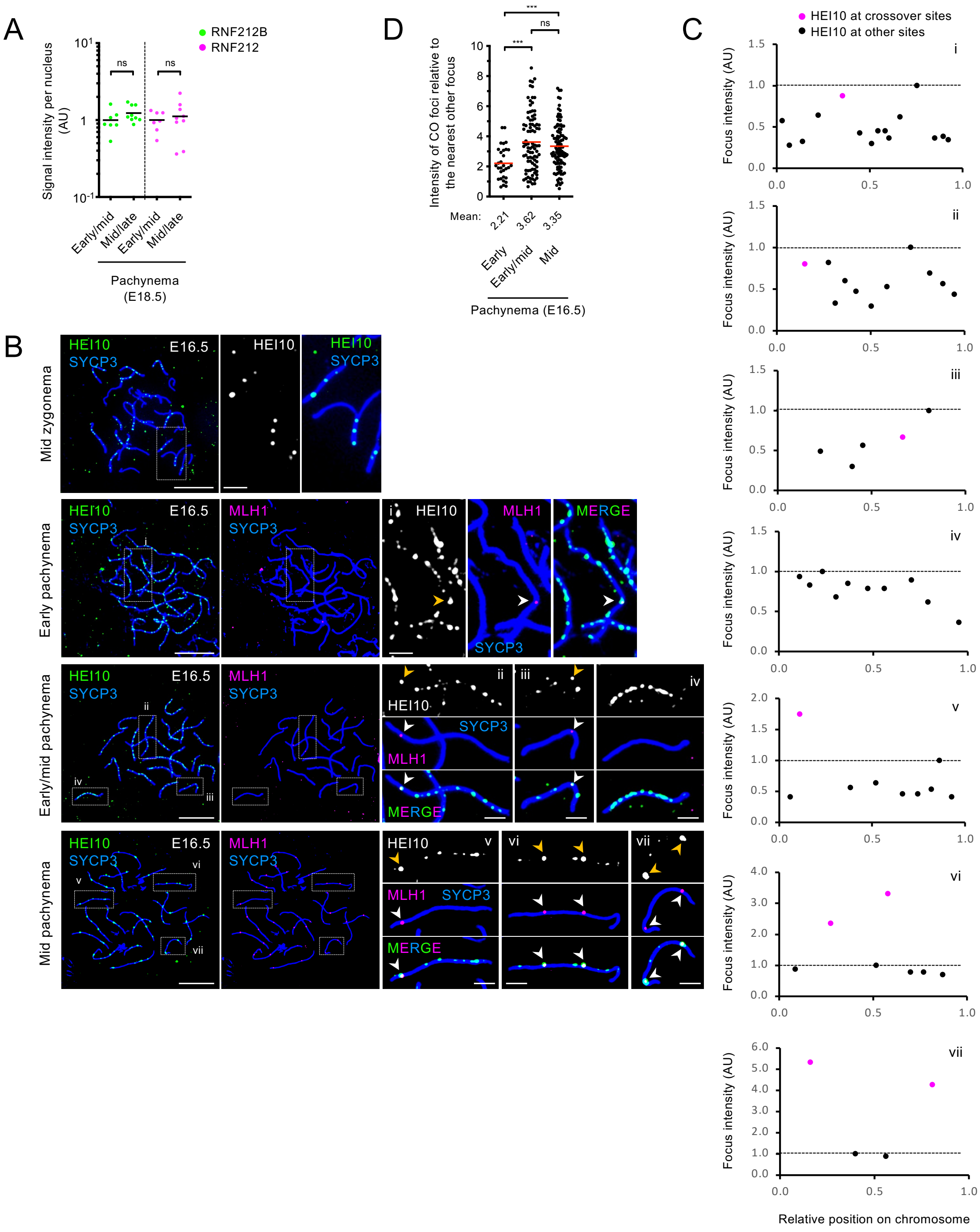

Fig. S14. Sexually dimorphic chromosomal localization of RNF212B, RNF212 and HEI10.
(A) Total signal intensity of RNF212B and RNF212 per nucleus in early/mid and mid/late pachytene oocytes. Black bars indicate means. ns, not significant ($p > 0.05$) for two-tailed Mann-Whitney tests. Nuclei analyzed were the same as in **Figure 6D**.
(B) HEI10 localization at successive prophase-I stages of fetal oocyte nuclei from embryonic day 16.5 (E16.5). Surface-spread prophase-I oocyte nuclei in mid zygonema, early pachynema, early/mid pachynema, and mid pachynema were immunostained for SYCP3, HEI10 and MLH1.

Arrowheads indicate crossover foci marked by MLH1. The magnified images show representative chromosomal regions. Scale bars, 10 μm for images of full nuclei and 2 μm for magnified panels.
(C) Representation of per chromosome analysis of HEI10 for pachytene chromosomes shown in (B). Each HEI10 focus is represented by a dot. Magenta dots indicate HEI10 foci at crossover sites (colocalized with MLH1 foci). The dashed line indicates the signal intensity of the brightest other focus (noncrossover focus) along the same chromosome.
(D) Per chromosome analysis for HEI10 focus intensity showing brighter HEI10 foci at crossover sites than adjacent foci. Intensity of each crossover-associated HEI10 focus relative to that of the nearest other (non-crossover) focus along the same chromosome. Red bars indicate means. Mean intensities are indicated below the graph. Detailed representation of per chromosome analysis is shown in (C). ns, not significant ($p > 0.05$); ***$p \leq 0.001$ for two-tailed Mann-Whitney tests. 31, 91, and 105 crossover foci from 14 early pachytene, 9 early/mid pachytene, and 6 mid pachytene nuclei were analyzed, respectively.

Table S1. Mutations and phenotypes of *Rnf212b* mutant lines generated in this study.

Founder mice with mono-allelic mutation

ID	Mutation		Homozygous mutant male
1E	1 nt insertion after 26 nt from first ATG	/ a premature stop codon after 27 a.a.	Infertile, small testes
2Q	1 nt insertion after 26 nt from first ATG	/ a premature stop codon after 27 a.a	Infertile, small testes
3K	1 nt substitution after 23 nt from first ATG	/ no change in coding a.a.;	Infertile, small testes
	3 nt deletion after 24 nt from first ATG	/ C9- mutation	
4A	10 nt deletion after 23 nt from first ATG	/ a premature stop codon after 27 a.a.	Infertile, small testes
5C	1 nt insertion after 27 nt from first ATG	/ a premature stop codon after 27 a.a.	Infertile, small testes
6U	8 nt insertion after 26 nt from first ATG	/ a premature stop codon after 8 a.a.	Infertile, small testes
7W	1 nt insertion after 26 nt from first ATG	/ a premature stop codon after 8 a.a.	Infertile, small testes

Founder mice with bi-allelic or chimeric mutation

ID	Allele	Mutation	Founder mouse
8M	1	2 nt deletion after 25 nt from first ATG / a premature stop codon after 26 a.a.	Infertile, small testes
	2	2 nt deletion followed by 1 nt substitution after 26 nt from first ATG / a premature stop codon after 26 a.a.	
9P	1	1-5-1 nt deletion after 16 nt from first ATG / a premature stop codon after 28 a.a.	Infertile, small testes
	2	2 nt insertion after 27 nt from first ATG / a premature stop codon after 31 a.a.	
	3	3 nt insertion followed by 1 nt substitution after 25 nt from first ATG	
10G	1	1 nt insertion after 26 nt from first ATG / a premature stop codon after 27 a.a.	Infertile (female)
	2	354 nt insertion after 25 nt from first ATG	

11I	1	9 nt deletion after 22 nt from first ATG / Q8-, C9-, F10- mutation	Infertile (female)
	2	12 nt deletion after 27 nt from first ATG / F10-, R11-, K12-, D13- mutation;<br>1 nt substitution after 61 nt from first ATG / S21N mutation	
12Y	1	2 nt deletion after 25 nt from first ATG / a premature stop codon after 26 a.a.	Infertile (female)
	2	4 nt deletion after 22 nt from first ATG / a premature stop codon after 29 a.a.	

Table S2. Primers used in this study.

Primer	Sequence (5'->3')
Cloning of *Rnf212b* gene to identify mutation, forward	GTTCTCTGGCATATAGTGAAG
Cloning of *Rnf212b* gene to identify mutation, reverse	CCCGACACAACTCAAAAGAC
Genotyping of *Rnf212b* wild-type allele, forward	TGGTTTCATTGCAACCAGTGT
Genotyping of *Rnf212b* null mutant allele, forward	TGGTTTCATTGCAACCAGTGG
Genotyping of *Rnf212b* RING mutant allele, forward	GGTTTCATTGCAACCAATTCC
Genotyping of *Rnf212b*, common reverse	CCCGACACAACTCAAAAGAC
RT-PCR for *Rnf212b* 5'UTR variant a, forward	CTGCTTCTCAAATGTCTATAAAG
RT-PCR for *Rnf212b* 5'UTR variant b, forward	CTGCTTCTCAAATGTAACAAGG
RT-PCR for *Rnf212b* 5'UTR variants a/b/c, forward	CTGCCAAGCTGAATGGATTG
RT-PCR for *Rnf212b* 5'UTR variants, common reverse	GAGAAGGGGTTTCAGTGTAAG
RT-PCR for *Rnf212b* isoforms a, b, c, forward	CTTTGGAAAAATGTGCTGTTTG
RT-PCR for *Rnf212b* isoform d, forward	CACATCTCTCAGTAGGTGTG
RT-PCR for *Rnf212b* isoforms a, d, reverse	GAGAAGGGGTTTCAGTGTAAG
RT-PCR for *Rnf212b* isoform b, reverse	GAGTCTTTCAGGCAGTGTAAG
RT-PCR for *Rnf212b* isoform c, reverse	TGGAGAAGGGGTTTCTGTTC
Cloning of *Rnf212b* cDNA into pGADT7, forward	ATGGCCATGGAGGCCAGTGAATTCATGGATTGGTTTCATTGCAACCAG
Cloning of *Rnf212b* cDNA into pGADT7, reverse	TGCAGCTCGAGCTCGATGGATCCCTATCTGGAAATGTTCCATCCGTCTTT
Cloning of *Rnf212b* cDNA into pGBKT7, forward	CATATGGCCATGGAGGCCGAATTCATGGATTGGTTTCATTGCAACCAG
Cloning of *Rnf212b* cDNA into pGBKT7, reverse	GGCCGCTGCAGGTCGACGGATCCCTATCTGGAAATGTTCCATCCGTCTTT

Cloning of *Rnf212* cDNA into pGADT7, forward	ATGGCCATGGAGGCCAGTGAATTCATGGCCAGCTGGGTGTTCTGTAAT
Cloning of *Rnf212* cDNA into pGADT7, reverse	TGCAGCTCGAGCTCGATGGATCCTCATCGATGAACGCATGCATGCCAGGG
Cloning of *Rnf212* cDNA into pGBKT7, forward	CATATGGCCATGGAGGCCGAATTCATGGCCAGCTGGGTGTTCTGTAAT
Cloning of *Rnf212* cDNA into pGBKT7, reverse	GGCCGCTGCAGGTCGACGGATCCTCATCGATGAACGCATGCATGCCAGGG
Point mutagenesis for *Rnf212b*^{C9A}	TTGGTTTCATTGCAACCAGGCTTTCCGAAAAGATGGGGCC
Point mutagenesis for *Rnf212*^{C10A}	GGTGTTCTGTAATCGCGCTTTCCAGTCGCCGCAC

Table S3. Antibodies used in this study.

Antibody	Source	Identifier	Application	Dilution
Guinea pig anti-RNF212B	this study	N/A	IF	1:40 (SIM, 1:20)
			IB	1:500
Goat anti-RNF212	Bondarieva et al., 2020 (11)	N/A	IF	1:50 (SIM, 1:25)
			IB	1:500
Guinea pig anti-RNF212	this study	N/A	IB	1:1,000
Mouse anti-SYCP3	Santa Cruz Biotechnology	sc-74569	IF	1:200 (SIM, 1:50)
Goat anti-SYCP3	Santa Cruz Biotechnology	sc-20845	IF	1:200 (SIM, 1:100)
			IB	1:1,000
Rabbit anti-SYCP3	Santa Cruz Biotechnology	sc-33195	IF	1:200
Rabbit anti-SYCP1	Abcam	ab15090	IF	1:200
Mouse anti-MLH1	BD Pharmingen	550838	IF (spermatocyte)	1:50
Mouse anti-MLH1	Cell Signaling Technology	3515S	IF (oocyte)	1:30
Rabbit anti-HEI10 (serum)	Qiao et al., 2014 (12)	N/A	IF	1:500
Mouse anti-CDK2	Santa Cruz Biotechnology	sc-6248	IF	1:100
Guinea pig anti-PRR19	a gift from Attila Tóth	N/A	IF	1:200
Rabbit anti-MSH4	a gift from Paula Cohen	N/A	IF	1:200
Guinea pig anti-TEX11	a gift from Christer Höög	N/A	IF	1:200
Rabbit anti-MER3	Rao et al., 2017 (13)	N/A	IF	1:200
Rabbit anti-RPA2	Abcam	ab76420	IF	1:200

Rabbit anti-DMC1	Santa Cruz Biotechnology	sc-22768	IF	1:50
Mouse anti-RAD51	ThermoScientific	MS-988-P0	IF	1:50
Guinea pig anti-H1t	a gift from Marry A. Handel	N/A	IF	1:1,000
Human anti-centromere	ImmunoVision	HCT-0100	IF	1:500
Rabbit anti-histone H3	Abcam	ab18521	IB	1:10,000
Mouse anti-p63	Santa Cruz Biotechnology	sc-25268	IF	1:500
Goat anti-mouse 488	Invitrogen	A-11029	IF	1:1,000
Goat anti-rabbit 488	Invitrogen	A-11070	IF	1:1,000
Goat anti-mouse 594	Invitrogen	A-11020	IF	1:2,000
Goat anti-rabbit 568	Invitrogen	A-11036	IF	1:1,000
Goat anti-human 555	Invitrogen	A-21433	IF	1:1,000
Goat anti-mouse 350	Invitrogen	A-21049	IF	1:50
Donkey anti-guinea pig 488	JacksonImmunoResearch	706-545-148	IF	1:100
Donkey anti-goat 488	JacksonImmunoResearch	705-545-147	IF	1:100
Donkey anti-goat Cy3	JacksonImmunoResearch	705-165-147	IF	1:100
Donkey anti-rabbit Cy3	JacksonImmunoResearch	711-165-152	IF	1:100
Donkey anti-rabbit 594	JacksonImmunoResearch	711-585-152	IF	1:500
Donkey anti-mouse 594	JacksonImmunoResearch	715-585-151	IF	1:100
Donkey anti-rabbit 647	JacksonImmunoResearch	711-605-152	IF	1:100
Donkey anti-guinea pig 647	JacksonImmunoResearch	706-605-148	IF	1:100
Donkey anti-goat 647	JacksonImmunoResearch	705-605-147	IF	1:100

Donkey anti-mouse AMCA	JacksonImmunoResearch	715-155-151	IF	1:50
Donkey anti-rabbit AMCA	JacksonImmunoResearch	711-155-152	IF	1:50
Donkey anti-goat AMCA	JacksonImmunoResearch	705-155-147	IF	1:50
Goat anti-guinea pig IgG(H+L)-HRP	Southern Biotech	6090-05	IB	1:1,000
Rabbit anti-goat IgG(H+L)-HRP	Southern Biotech	6020-05	IB	1:1.000

IF, immunofluorescence; IB, immunoblotting.

SI References

1. A. Reynolds *et al.*, RNF212 is a dosage-sensitive regulator of crossing-over during mammalian meiosis. *Nat Genet* **45**, 269-278 (2013).
2. J. O. Ward *et al.*, Mutation in mouse hei10, an e3 ubiquitin ligase, disrupts meiotic crossing over. *PLoS Genet* **3**, e139 (2007).
3. F. Baudat, K. Manova, J. P. Yuen, M. Jasin, S. Keeney, Chromosome synapsis defects and sexually dimorphic meiotic progression in mice lacking Spo11. *Molecular cell* **6**, 989-998 (2000).
4. F. A. de Vries *et al.*, Mouse Sycp1 functions in synaptonemal complex assembly, meiotic recombination, and XY body formation. *Genes Dev* **19**, 1376-1389 (2005).
5. S. M. Lipkin *et al.*, Meiotic arrest and aneuploidy in MLH3-deficient mice. *Nat Genet* **31**, 385-390 (2002).
6. A. R. Bassett, C. Tibbit, C. P. Ponting, J. L. Liu, Highly efficient targeted mutagenesis of Drosophila with the CRISPR/Cas9 system. *Cell Rep* **4**, 220-228 (2013).
7. A. H. Peters, A. W. Plug, M. J. van Vugt, P. de Boer, A drying-down technique for the spreading of mammalian meiocytes from the male and female germline. *Chromosome Res* **5**, 66-68 (1997).
8. M. Ito *et al.*, FIGNL1 AAA+ ATPase remodels RAD51 and DMC1 filaments in pre-meiotic DNA replication and meiotic recombination. *Nat Commun* **14**, 6857 (2023).
9. Y. Yun, M. Ito, S. Sandhu, N. Hunter, Cytological Monitoring of Meiotic Crossovers in Spermatocytes and Oocytes. *Methods Mol Biol* **2153**, 267-286 (2021).
10. M. Di Giacomo *et al.*, Distinct DNA-damage-dependent and -independent responses drive the loss of oocytes in recombination-defective mouse mutants. *Proc Natl Acad Sci U S A* **102**, 737-742 (2005).
11. A. Bondarieva *et al.*, Proline-rich protein PRR19 functions with cyclin-like CNTD1 to promote meiotic crossing over in mouse. *Nat Commun* **11**, 3101 (2020).
12. H. Qiao *et al.*, Antagonistic roles of ubiquitin ligase HEI10 and SUMO ligase RNF212 regulate meiotic recombination. *Nat Genet* **46**, 194-199 (2014).
13. H. B. Rao *et al.*, A SUMO-ubiquitin relay recruits proteasomes to chromosome axes to regulate meiotic recombination. *Science* **355**, 403-407 (2017).
